# Neuroinflammation in postoperative cognitive dysfunction: the multi-target potential of esketamine in modulating microglial responses and synaptic integrity

**DOI:** 10.3389/fnmol.2026.1868647

**Published:** 2026-09-02

**Authors:** Hao-Tian Li, Guang-Yong Lin, Wen-Tao Xing, Yu Liu, Chuan-Xiang Zhang

**Affiliations:** 1 Department of Anesthesiology, Wuxi Xinwu District Xinrui Hospital, Wuxi, Jiangsu, China; 2 Department of Anesthesiology, The Second Hospital of Chifeng, Chifeng, Inner Mongolia, China; 3 Department of Anesthesiology, The Third People’s Hospital of Longgang, Clinical Institute of Shantou University Medical College (The Third People’s Hospital of Longgang District Shenzhen), Shenzhen, Guangdong, China

**Keywords:** autophagy, esketamine, microglial responses, neuroinflammation, perioperative neurocognitive disorders, perioperative neuroprotection, postoperative cognitive dysfunction, synaptic plasticity

## Abstract

Postoperative cognitive dysfunction (POCD) remains a clinically important problem after surgery, particularly in older and neurologically vulnerable patients. Its interpretation is complicated by heterogeneous cognitive definitions and follow-up periods, while the underlying biology appears to involve interacting inflammatory, metabolic, glial, and synaptic disturbances rather than a single pathogenic cascade. Esketamine has therefore attracted interest as a potential perioperative neuroprotective agent, but the strength of evidence differs markedly across the mechanisms proposed to explain its effects. We conducted a structured narrative review with evidence mapping of clinical and experimental studies identified in PubMed/MEDLINE and the Web of Science Core Collection, using a literature cutoff of 1 May 2026. Direct perioperative clinical and preclinical studies were distinguished from non-perioperative esketamine research, ketamine/enantiomer studies, and contextual POCD/PND biology, with greater mechanistic weight assigned to experiments incorporating pathway perturbation or rescue. The direct preclinical literature favors a multi-branch model of esketamine action. Functional evidence supports contributions from TLR4/MyD88–p38 signaling, STING/TBK1-associated inflammatory cell death, and PARP1-related autophagic regulation, while changes in NF-κB signaling, microglial BDNF–TrkB activity, and ROCK2/ADD1-associated synaptic remodeling are supported mainly by convergent molecular and functional findings. NLRP3 remains relevant to postoperative neuroinflammation but has not been established as a required upstream mediator of esketamine action, and proposed effects on the blood–brain barrier, antioxidant pathways, cholinergic signaling, and neurogenesis remain less directly supported in perioperative cognitive models. Clinical findings are less uniform. Some randomized trials suggest reductions in postoperative delirium or early postoperative cognitive decline in selected populations, whereas other adequately designed studies are neutral, and evidence for sustained cognitive protection is limited. Peripheral inflammatory, neuronal-injury, and neurotrophic biomarkers indicate biological activity but do not establish central target engagement or cognitive mediation. Taken together, the current literature supports a biologically plausible, multi-target model for esketamine, but not an established preventive effect against perioperative neurocognitive disorders.

## 1 Introduction

Cognitive decline after surgery remains a major concern in older and neurologically vulnerable patients. The term has historically been used to describe postoperative deterioration in memory, attention, executive function, processing speed, or other cognitive domains relative to preoperative performance. Yet the reported incidence and persistence of these deficits vary widely across surgical populations and, importantly, across the methods used to define them (van Sinderen et al., 2020). Brief cognitive screening, formal neuropsychological testing, postoperative delirium (POD), and longer-term cognitive decline do not measure the same clinical construct. Contemporary perioperative neurocognitive disorder (PND) nomenclature provides a more structured framework for these distinctions (Evered et al., 2018), although much of the existing mechanistic and interventional literature continues to use postoperative cognitive dysfunction (POCD). We therefore retain POCD when referring to that legacy literature, while analyzing POD separately as an acute postoperative syndrome and distinguishing delayed neurocognitive recovery (dNCR), later postoperative cognitive decline, and isolated changes in screening scores where the original studies permit.

Persistent postoperative cognitive decline can have consequences beyond the immediate recovery period; earlier ISPOCD follow-up linked postoperative cognitive dysfunction with adverse long-term outcomes, including increased mortality and premature withdrawal from the labor market (Steinmetz et al., 2009). Its relationship to subsequent dementia, however, requires more cautious interpretation. In a later follow-up of patients from the International Study of Postoperative Cognitive Dysfunction cohorts, POCD identified at 1 week or 3 months was not significantly associated with subsequently registered dementia over a median follow-up of approximately 11 years (Steinmetz et al., 2013). Thus, although postoperative cognitive deterioration may identify a vulnerable patient population, current evidence does not justify presenting POCD as an established cause or independent predictor of later dementia. This distinction is especially relevant in older adults, in whom surgery occurs against a background of highly variable cognitive reserve, vascular burden, systemic inflammation, and pre-existing neuropathology.

The biological response to surgery is similarly difficult to reduce to a single pathway. Tissue injury initiates systemic inflammatory and metabolic signals that communicate with the central nervous system through neurovascular, humoral, and cellular routes (Subramaniyan and Terrando, 2019). Within the brain, innate immune signaling intersects with glial regulation, oxidative and mitochondrial stress, cellular homeostasis, and synaptic function (Yang et al., 2020). These processes evolve over time and are shaped by the host as well as the surgical insult. Neuroinflammation is therefore better regarded as an interacting pathological environment than as a fixed sequence in which one upstream pathway necessarily determines postoperative cognitive outcome.

Esketamine has attracted interest in this setting because its actions extend beyond N-methyl-D-aspartate receptor antagonism. Direct perioperative experiments have linked esketamine exposure to inflammatory signaling through STING/TBK1 and TLR4/MyD88–p38 pathways (Li et al., 2022; Xu et al., 2024), while perturbation-based evidence also implicates PARP1-related autophagic regulation in cellular stress homeostasis (Zhao et al., 2025). Changes in microglial responses, BDNF–TrkB signaling, and synaptic plasticity have likewise been observed in perioperative models, although pathway necessity has not been established to the same extent (Wen et al., 2024). This unequal mechanistic depth is mirrored clinically. A randomized trial using multidomain neuropsychological assessment found less dNCR at 7 days but no significant difference in POCD at 3 months (Han et al., 2023), whereas a larger factorial randomized trial found no significant reduction in POD or improvement in MMSE with esketamine (Zhang et al., 2024). The resulting question is not whether a plausible neuroprotective mechanism can be proposed, but which mechanisms are directly supported in perioperative cognitive models, how much causal weight they can bear, and whether those experimental effects correspond to clinically meaningful cognitive benefit.

## 2 Methods: literature search and evidence synthesis

### 2.1 Review design and search strategy

This study was conducted as a structured narrative review with evidence mapping. The review addressed clinical studies of perioperative esketamine/S-ketamine and postoperative neurocognitive outcomes, together with experimental studies relevant to the mechanisms underlying these effects.

PubMed and the Web of Science Core Collection were searched, with eligibility restricted to records published or available online on or before 1 May 2026. The searches were executed on 7 August 2026. Three complementary search streams were used. Search A identified clinical studies of perioperative esketamine/S-ketamine reporting postoperative neurocognitive outcomes; Search B identified mechanistic studies of esketamine relevant to perioperative neuroinflammation, glial responses, cellular stress, autophagy, mitochondrial function, neurovascular regulation, and synaptic plasticity; and Search C covered broader POCD/PND pathophysiology. Searches A and B formed the main evidence-selection track, whereas Search C was used only to provide disease-mechanism context. The complete database-specific search strategies are provided in Supplementary Table 1.

### 2.2 Eligibility and study selection

Human studies were eligible for the clinical evidence synthesis when they evaluated perioperative esketamine/S-ketamine and reported a postoperative neurocognitive outcome. Outcomes included POD, dNCR, other study-defined postoperative cognitive decline, longer-term postoperative cognitive outcomes, and postoperative cognitive test scores. Studies reporting only non-cognitive perioperative outcomes were not considered primary evidence of cognitive efficacy.

Direct preclinical studies were eligible when esketamine/S-ketamine was evaluated in a surgery- or anesthesia-related cognitive model with behavioral and/or relevant mechanistic outcomes. Esketamine studies outside the perioperative cognitive setting, studies of racemic ketamine or other enantiomers, and general POCD/PND mechanistic studies were retained only as indirect or contextual evidence. Reviews, editorials, protocols, letters, bibliometric studies, conference abstracts without sufficient primary data, duplicate or overlapping publications without unique data, and records beyond the eligibility cutoff were excluded from the primary synthesis.

**FIGURE 1:**
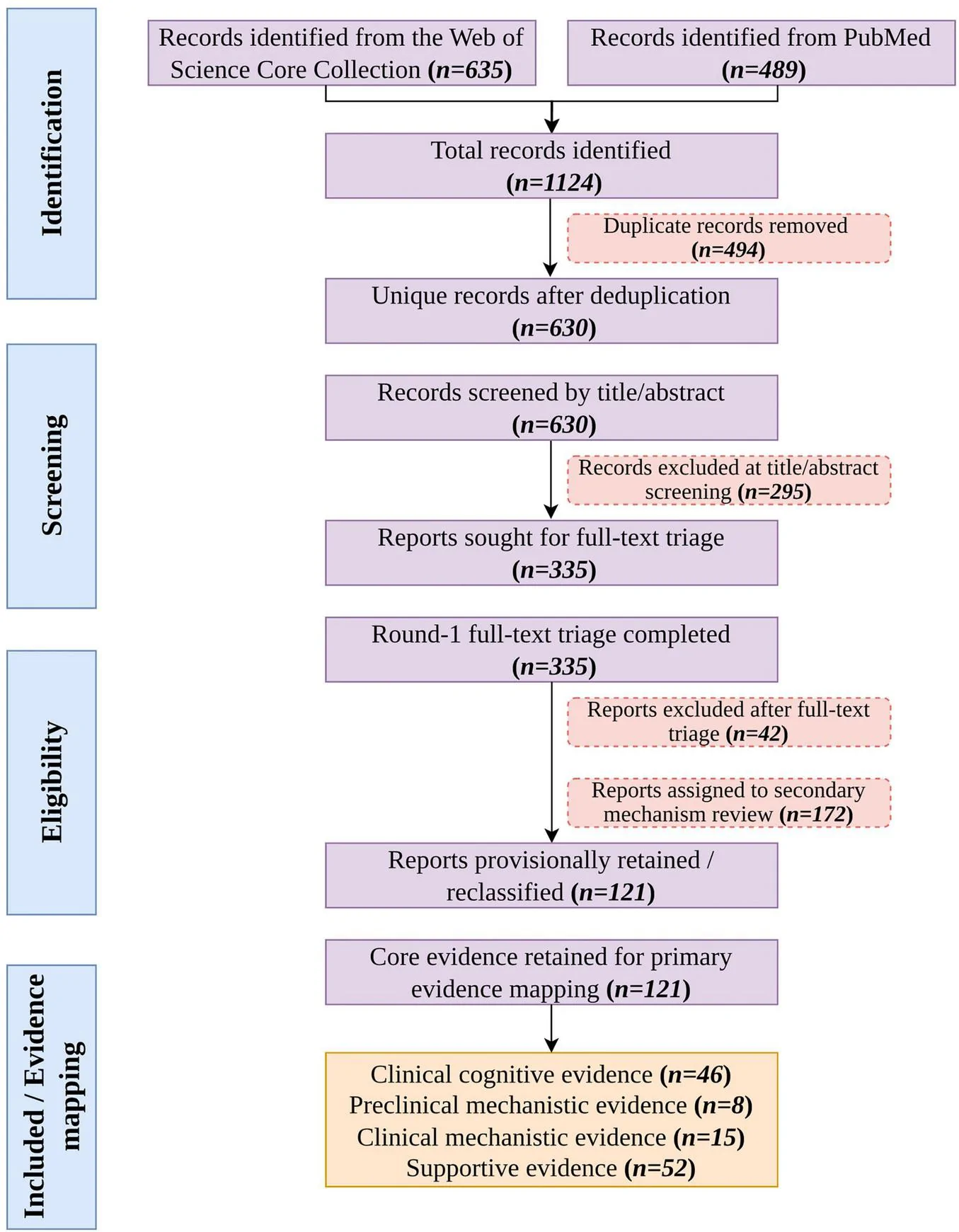
PRISMA-style flow diagram of study identification, screening, and evidence mapping. Records were identified through PubMed and the Web of Science Core Collection using the prespecified clinical (Search A) and esketamine mechanistic (Search B) search strategies. After deduplication, records underwent title/abstract screening followed by round-1 full-text triage. Records assigned to the secondary mechanism review were not retained as core direct perioperative evidence at this stage but were considered potentially informative for prespecified mechanisms, principally as indirect esketamine or ketamine/enantiomer evidence. These records were reviewed separately for mechanistic relevance and did not contribute to the core evidence count. The 121 records retained for primary evidence mapping comprised clinical cognitive evidence (*n* = 46), preclinical mechanistic evidence (*n* = 8), clinical mechanistic evidence (*n* = 15), and supportive evidence (*n* = 52). Search C, which covered broader POCD/PND pathophysiology, was used separately for contextual disease-mechanism mapping and was not included in the main PRISMA-style flow. The literature eligibility cutoff was 1 May 2026, and searches were executed on 7 August 2026.

Records were deduplicated using DOI, PMID, Web of Science accession number, normalized title, and bibliographic metadata. Screening was performed at the title/abstract and full-text levels. Reports originating from the same participant cohort or experimental study were linked at the study level to avoid double counting (Page et al., 2021). The study-selection process, including deduplication, title/abstract screening, full-text triage, and allocation to the core and secondary mechanistic evidence streams, is summarized in Figure 1.

### 2.3 Data extraction and evidence classification

For clinical studies, we extracted study design and population, sample size, esketamine regimen and comparator, neurocognitive outcome, assessment instrument or diagnostic definition, timing of assessment, principal findings, reported mechanistic biomarkers, and major limitations. POD, dNCR, longer-term postoperative cognitive decline, and isolated changes in screening scores were retained as separate outcome categories. In particular, changes in Mini-Mental State Examination (MMSE) or Montreal Cognitive Assessment (MoCA) scores were not considered equivalent to formally defined postoperative neurocognitive disorders (Evered et al., 2018).

For preclinical studies, extracted variables included the perioperative model, esketamine exposure, proposed mechanistic pathway, molecular or cellular readouts, pathway-specific perturbation or rescue, and cognitive or functional outcomes. Clinical inflammatory, neuronal-injury, and neurotrophic biomarkers were treated as mechanistic correlates unless central target engagement or mediation of the cognitive outcome had been directly examined.

Evidence was classified as direct clinical perioperative evidence, direct perioperative preclinical evidence, indirect esketamine mechanistic evidence, ketamine/enantiomer mechanistic evidence, or contextual POCD/PND evidence. Within the preclinical literature, pathway perturbation, pharmacological reversal, or genetic manipulation was assigned greater mechanistic weight than parallel changes in molecular markers and behavior alone; exploratory transcriptomic or network-based findings were regarded as hypothesis-generating until functionally validated.

### 2.4 Evidence synthesis

Clinical findings were synthesized according to the neurocognitive outcome assessed rather than under a single composite category. POD was considered a clinically relevant but distinct acute outcome and was not used as a surrogate for dNCR or longer-term postoperative cognitive decline. Positive and neutral randomized trials were considered together, and studies in which esketamine formed part of a multimodal anesthetic or analgesic regimen were interpreted with greater caution regarding drug-specific attribution.

Mechanistic conclusions were weighted according to directness and experimental support. Findings from non-perioperative esketamine models, ketamine/enantiomer studies, and general POCD pathophysiology were used to assess biological plausibility but did not increase the causal strength of perioperative esketamine-specific claims.

Because the clinical studies differed substantially in surgical population, esketamine regimen, comparator, cognitive definition, assessment instrument, and follow-up interval, quantitative pooling was not undertaken. The evidence was synthesized qualitatively, with interpretation guided by outcome definition, treatment attribution, mechanistic directness, and causal strength.

## 3 Pathophysiological context of postoperative cognitive dysfunction

Postoperative cognitive decline emerges from the interaction between the surgical insult and the biological susceptibility of the brain exposed to it. Surgical injury generates systemic immune and metabolic responses, but their neurological consequences depend on how those signals are communicated across the neurovascular interface, interpreted by resident brain cells, and ultimately translated into disturbed neuronal and synaptic function (Subramaniyan and Terrando, 2019). The following section therefore provides the pathological background for the esketamine-specific evidence considered in section “4 Multi-target neuroprotective mechanisms of esketamine in postoperative cognitive dysfunction”; most of this disease framework derives from the broader POCD/PND literature rather than from intervention studies of esketamine itself.

### 3.1 Peripheral injury and neurovascular signaling

Tissue injury releases damage-associated molecular patterns (DAMPs), including high-mobility group box 1 (HMGB1) and mitochondrial DNA, accompanied by a marked postoperative cytokine response involving mediators such as IL-6 and TNF-α (Leijte et al., 2018). Purinergic signaling has also been implicated in the propagation of surgery-induced neuroinflammation, with inhibition or deletion of P2 * 7 receptors attenuating postoperative inflammatory and cognitive abnormalities in mice (Zheng et al., 2017). The magnitude of the systemic response depends on surgical stress and patient characteristics, and circulating cytokine concentrations should not be taken as direct measures of neuroinflammation.

Peripheral inflammation can nevertheless influence the brain without requiring a simple model in which circulating cytokines pass directly into the central nervous system. In aged rats, surgery was associated with increased systemic and hippocampal inflammatory signaling, HMGB1/RAGE upregulation, BBB disruption, and postoperative learning impairment (He et al., 2012). MMP-9 provides another experimentally supported route linking peripheral inflammation to altered BBB permeability: surgery-induced BBB disruption, neuroinflammation, and cognitive impairment were markedly attenuated in MMP9-deficient mice (Bi et al., 2017). Its contribution is likely context dependent, however, and direct evidence relating the magnitude of BBB disruption to postoperative cognitive phenotype in patients remains limited. BBB dysfunction is therefore relevant to POCD pathophysiology without being an obligatory first event in every model—or, by extension, a proven primary target of esketamine.

### 3.2 Central immune and cellular-stress responses

Once perioperative signals reach or influence the brain, microglia, astrocytes, endothelial cells, and neurons participate in a response that is both inflammatory and metabolic. Experimental perioperative cognitive models demonstrate that mitochondrial dysfunction and oxidative stress can occur alongside neuroinflammation, synaptic abnormalities, and learning and memory impairment (Zuo et al., 2020). Autophagic homeostasis may also be disturbed after anesthesia and surgery, with changes in mTOR signaling accompanied by reduced synaptic proteins and impaired memory (Li et al., 2022). These processes are interconnected rather than sequential: mitochondrial stress can generate reactive oxygen species and additional danger signals, inflammatory signaling can modify cellular metabolism, and impaired cellular quality control may prolong tissue dysfunction after the initial systemic stimulus has diminished.

TLR-dependent inflammatory signaling and inflammasome-related pathways are among the most frequently investigated components of this response. NLRP3 has substantial experimental support in PND/POCD models. In aged surgical mice, selective NLRP3 inhibition with MCC950 suppressed hippocampal neuroinflammation and attenuated postoperative cognitive deficits (Fu et al., 2020). Mitochondrial dysfunction has also been associated with NLRP3–caspase-1-dependent pyroptotic signaling, loss of synaptic integrity, and impaired learning and memory after anesthesia and surgery (Zuo et al., 2020). This background establishes NLRP3 as a biologically relevant component of perioperative neuroinflammation, but not as a treatment-specific mediator of any particular intervention. Other innate immune and cellular-stress pathways can converge on overlapping inflammatory and cell-injury outputs. Accordingly, NLRP3 is treated here as one node within the postoperative inflammatory landscape rather than as the obligatory upstream mediator through which esketamine must act.

Ageing may magnify several elements of this response simultaneously. In direct comparisons, older rats showed greater susceptibility to surgery-induced neuroinflammation and cognitive dysfunction than younger animals, with age-related impairment of TAK1/RIPK1 regulation contributing to this vulnerability (Zhang Y. et al., 2023). Mitochondrial stress regulation also appears relevant: hippocampal SIRT3 restoration in aged mice reduced oxidative stress and neuroinflammation while improving postoperative cognitive and synaptic phenotypes (Liu Q. et al., 2021). These findings support the broader view that ageing lowers the capacity to restore neural homeostasis after surgical stress rather than creating a single age-specific pathogenic pathway.

### 3.3 Glial–synaptic coupling and cognitive dysfunction

The clinical relevance of neuroinflammation ultimately depends on its effect on neuronal communication. Microglial responses after surgery are heterogeneous and should not be reduced to a universal M1-to-M2 switch. In experimental POCD, microglia can participate directly in pathological synaptic remodeling. In aged mice, inhibition of microglial activity or disruption of complement C3 receptor signaling reduced the phagocytosis and loss of inhibitory synapses and improved postoperative cognitive performance (Tan et al., 2023). Astrocytic and other glial disturbances may further alter metabolic, transmitter, and trophic support, creating a less stable environment for synaptic transmission and plasticity.

Changes in synaptic structure and function are consequently a plausible convergence point between perioperative inflammation and cognitive impairment. Surgery and anesthesia have been associated with reduced synaptophysin and PSD-95, impaired hippocampal synaptic plasticity, and memory deficits in aged mice (Gao et al., 2021). A separate study linking mitochondrial oxidative stress and neuroinflammation to postoperative cognition showed that SIRT3 manipulation altered dendritic morphology and hippocampal long-term potentiation together with behavioral performance (Liu Q. et al., 2021). The hippocampus is particularly well studied because of its sensitivity to inflammatory and metabolic stress, although postoperative cognition is not exclusively hippocampal: attention, executive function, and processing speed depend on distributed cortical and subcortical networks that remain much less thoroughly characterized experimentally.

This broader view is important for interpreting intervention studies. Improvement in an inflammatory marker, a hippocampal protein, or a single behavioral assay does not by itself demonstrate comprehensive cognitive neuroprotection. More informative evidence links molecular change to cellular or synaptic recovery and, ideally, to behavioral function within the same perioperative model. Section “4 Multi-target neuroprotective mechanisms of esketamine in postoperative cognitive dysfunction” therefore narrows the question from what can contribute to postoperative cognitive dysfunction to what has actually been modified by esketamine, and with what level of causal support.

## 4 Multi-target neuroprotective mechanisms of esketamine in postoperative cognitive dysfunction

Mechanistic studies of esketamine in POCD increasingly support a network-based model rather than a single dominant inflammatory pathway. The strength of evidence, however, varies substantially across the proposed mechanisms. Direct perioperative studies have implicated TLR4-dependent inflammatory signaling, autophagic and mitochondrial homeostasis, STING/TBK1-associated pyroptotic responses, microglial–neurotrophic interactions, and pathways related to synaptic structural integrity. By contrast, several mechanisms frequently invoked to explain the neuroprotective actions of esketamine—including direct NLRP3 inflammasome inhibition, specific BBB-protective signaling, and α7-nicotinic acetylcholine receptor (α7-nAChR) activation—derive predominantly from contextual POCD studies or from esketamine models outside the perioperative setting.

Accordingly, this section gives priority to studies in which esketamine was tested directly in surgery- or anesthesia-related cognitive models. Greater mechanistic weight is assigned to experiments incorporating pathway perturbation or pharmacological reversal than to studies showing parallel changes in signaling proteins and behavioral outcomes alone.

### 4.1 TLR4-dependent inflammatory signaling: NF-κB and p38 MAPK

TLR4-dependent signaling currently represents one of the better-supported inflammatory mechanisms linking esketamine to postoperative cognitive protection. In aged mice exposed to acute sleep deprivation before surgery, postoperative esketamine improved learning and memory and reduced hippocampal injury and inflammatory cytokine production. At the molecular level, esketamine decreased hippocampal TLR4 and MyD88 expression and reduced NF-κB p65 phosphorylation. Double-label immunofluorescence further demonstrated reduced phosphorylated p65 immunoreactivity in Iba1-positive microglia (Han et al., 2026). These findings directly associate attenuation of TLR4/MyD88/NF-κB signaling with improvement of surgery-induced cognitive deficits, although neither TLR4 nor NF-κB was selectively manipulated in this study. The data therefore support involvement of this pathway rather than establishing NF-κB as an indispensable mediator of esketamine’s effect.

A related perioperative study provides stronger functional evidence for the p38 MAPK branch. In aged mice undergoing surgery, esketamine improved Morris water maze performance, reduced hippocampal neuronal apoptosis, and lowered IL-1β, IL-6, and TNF-α in parallel with suppression of TLR4, MyD88, and p38 MAPK signaling. Importantly, pharmacological activation of p38 MAPK with anisomycin attenuated these behavioral, histological, and inflammatory improvements (Xu et al., 2024). Such reversal experiments provide stronger support for a functional contribution of the pathway than changes in protein abundance alone.

Rather than defining a single linear TLR4–NF-κB pathway, these studies suggest that esketamine attenuates a broader TLR4-centered inflammatory response. NF-κB and p38 MAPK represent partially overlapping downstream programs governing cytokine transcription, cellular stress, and neuronal injury. Their relative contribution is likely to vary with the perioperative insult and the biological vulnerability of the model (Xu et al., 2024; Han et al., 2026).

The relevance of this pathway is also supported by perioperative cognitive models using interventions other than esketamine. In aged mice undergoing splenectomy, suppression of microglial TLR4/MyD88/NF-κB signaling was associated with reduced neuroinflammation and cognitive impairment, whereas intracerebroventricular TLR4 activation weakened the protective effect of fluoxetine (Yao et al., 2023). A separate aged-mouse model of sevoflurane-induced cognitive dysfunction likewise showed attenuation of TLR4/NF-κB signaling together with reduced neuroinflammation and neuronal injury, with pharmacological TLR4 activation diminishing the intervention effect (Chen et al., 2025). These studies do not establish the mechanism of esketamine, but they strengthen the biological plausibility of TLR4-centered inflammatory signaling as a modifiable component of perioperative cognitive injury.

### 4.2 Cellular stress homeostasis: autophagy and mitochondrial regulation

Neuroinflammation after surgery is closely coupled to disturbances in intracellular stress handling. Recent experimental work suggests that esketamine may influence this interface through both autophagic regulation and mitochondrial homeostasis.

Zhao et al. examined the PARP1/SIRT1 axis in mice subjected to exploratory laparotomy and in LPS-stimulated BV2 microglial cells. Esketamine improved postoperative spatial learning and was accompanied by lower hippocampal concentrations of IL-1β, IL-6, and TNF-α, reduced PARP1 expression, increased SIRT1, and changes in LC3 and p62 consistent with enhanced autophagic activity (Zhao et al., 2025). In BV2 cells, PARP1 overexpression reproduced an inflammatory and autophagy-impaired phenotype characterized by increased inflammatory mediators, reduced SIRT1, altered LC3 and p62, lower NAD^+^ availability, and fewer autophagic structures. Esketamine partially reversed these changes, while rapamycin produced a broadly similar autophagy-associated anti-inflammatory phenotype (Zhao et al., 2025). These perturbation experiments strengthen the link between PARP1 regulation and esketamine-associated restoration of cellular homeostasis, although SIRT1 itself was not selectively inhibited or silenced. SIRT1-related findings from broader perioperative models provide further context. In aged rats exposed to anesthesia and surgery, restoration of SIRT1 activity was associated with reduced hippocampal tau acetylation and hyperphosphorylation, diminished inflammatory stress, and better cognitive performance (Yan et al., 2020). More recent work has linked SIRT1/FOXO1 signaling to autophagic regulation and oxidative-stress control in postoperative cognitive models (Zhang et al., 2025). These studies support a broader role for SIRT1 in postoperative cellular homeostasis, but they do not demonstrate that SIRT1 is required for the cognitive effect of esketamine.

The association between impaired autophagic homeostasis and postoperative cognitive dysfunction is not unique to the esketamine model. In aged mice, surgery was accompanied by mTOR hyperactivation, impaired hippocampal autophagy, reduced BDNF and synaptophysin, and postoperative memory deficits; rapamycin restored autophagic activity and improved behavioral performance (Jiang et al., 2020). A related study reported recovery of synaptophysin and PSD-95 together with cognitive improvement after mTOR inhibition and autophagy activation (Gao et al., 2021). These observations support autophagic dysfunction as a plausible component of perioperative neuronal stress, while leaving open which upstream regulator is most relevant to esketamine.

The biological relevance of this pathway extends beyond cytokine suppression. In the cellular experiments, PARP1 overexpression was accompanied by reduced NAD^+^ availability as well as impaired autophagic indices (Zhao et al., 2025), providing a potential link between PARP1 activity, metabolic stress, and intracellular quality control. Restoration of autophagic processing could in turn limit the persistence of damaged proteins and organelles and reduce sustained inflammatory signaling. In this setting, the PARP1/SIRT1 findings position intracellular quality control as a potential intermediate between surgical stress and neuroinflammatory amplification.

Mitochondrial SIRT3 has independently emerged as a regulator of perioperative cognitive vulnerability. Pharmacological activation of SIRT3 with honokiol reduced mitochondrial oxidative stress, neuroinflammation, neuronal apoptosis, and surgery-associated memory impairment, whereas the SIRT3 inhibitor 3-TYP weakened these effects (Ye et al., 2019). In aged mice, hippocampal AAV-mediated SIRT3 overexpression also attenuated oxidative stress, microglial inflammatory responses, synaptic plasticity abnormalities, and postoperative learning and memory deficits (Liu Q. et al., 2021). Similar findings have been reported in sevoflurane-related cognitive impairment following experimental SIRT3 upregulation (Dai et al., 2024).

A second line of evidence points toward mitochondrial regulation through SIRT3/AMPK/mTOR signaling. In aged mice subjected to abdominal surgery, esketamine improved spatial memory, reduced inflammatory and oxidative injury, attenuated markers associated with a pro-inflammatory microglial state, and improved indices of mitochondrial function (Wang et al., 2026). Importantly, the mechanistic perturbation was performed in parallel LPS-treated BV2 experiments rather than in the surgical animals. SIRT3 silencing attenuated esketamine-associated changes in AMPK/mTOR signaling, inflammatory markers, oxidative stress, mitochondrial membrane potential, and ATP-related indices (Wang et al., 2026). Evidence outside the perioperative cognitive setting also suggests that the relationship between esketamine, AMPK/mTOR signaling, autophagic regulation, and oxidative stress is not unique to the postoperative model. In experimental traumatic brain injury, esketamine promoted AMPK/mTOR-dependent TFEB nuclear translocation and autophagic activity while reducing oxidative neuronal injury; inhibition of AMPK attenuated several of these cellular effects (Tang et al., 2023). Because this evidence derives from traumatic brain injury rather than PND, it supports mechanistic plausibility rather than direct perioperative causality.

The PARP1/SIRT1 and SIRT3/AMPK/mTOR findings should not be interpreted as components of a single sirtuin pathway. They instead highlight two complementary dimensions of cellular stress regulation: autophagic quality control and mitochondrial metabolic resilience. Both may help restrain the transition from transient perioperative stress to sustained inflammatory and neuronal dysfunction.

### 4.3 STING/TBK1-associated pyroptosis and the contextual role of NLRP3

Among the mechanisms linking esketamine to inflammatory cell death, the strongest direct perioperative evidence currently involves STING/TBK1 signaling. In aged rats subjected to exploratory laparotomy, persistent postoperative cognitive deficits were accompanied by increased STING and TBK1 expression in the hippocampus and ventromedial prefrontal cortex, alterations in astrocytic phenotypes, and indices of mitochondrial dysfunction. In complementary cellular experiments, inflammatory stimulation reduced mitochondrial membrane potential and increased pyroptosis-associated proteins, including cleaved caspase-1 and IL-18. Esketamine reversed several of these abnormalities (Li et al., 2022).

The pharmacological manipulation of STING is particularly informative. Administration of the STING agonist ADU-S100 attenuated the protective effects of esketamine, whereas the STING antagonist C-176 further enhanced them (Li et al., 2022). These findings provide comparatively strong evidence that STING/TBK1 signaling contributes functionally to the postoperative phenotype and suggest a mechanistic connection between innate immune sensing, mitochondrial stress, and caspase-1-associated inflammatory cell death. The broader perioperative literature provides an additional mechanistic link between mitochondrial stress and STING signaling. In a sevoflurane-induced cognitive model, mitochondrial fission and cytosolic mtDNA release were associated with activation of the cGAS–STING pathway and downstream NLRP3 signaling; pharmacological cGAS blockade with RU.521 attenuated both inflammasome activation and cognitive dysfunction (Yang et al., 2024). This study is independent of esketamine, but it supports the biological connection among mitochondrial danger signaling, STING activation, and postoperative neuroinflammation suggested by the direct esketamine experiments.

NLRP3 remains relevant to the broader biology of perioperative cognitive dysfunction, but its position requires greater restraint. In aged perioperative models, mitochondrial dysfunction has been linked to NLRP3–caspase-1-dependent pyroptotic signaling, loss of synaptic integrity, and impaired cognition (Zuo et al., 2020), while selective NLRP3 inhibition with MCC950 attenuated hippocampal inflammatory responses and cognitive deficits after surgery (Fu et al., 2020). Other experimental models have connected reactive oxygen species to microglial NLRP3 activation and GSDMD-associated pyroptosis; both ROS scavenging and pyroptosis inhibition improved cognitive outcomes (Zhou et al., 2023). Genetic suppression of PYRIN-containing Apaf1-like protein 1, an earlier designation used in this experimental literature, likewise reduced inflammasome-associated signaling, microglial activation, caspase-1 activity, and isoflurane-induced cognitive impairment (Zhang et al., 2020).

**FIGURE 2:**
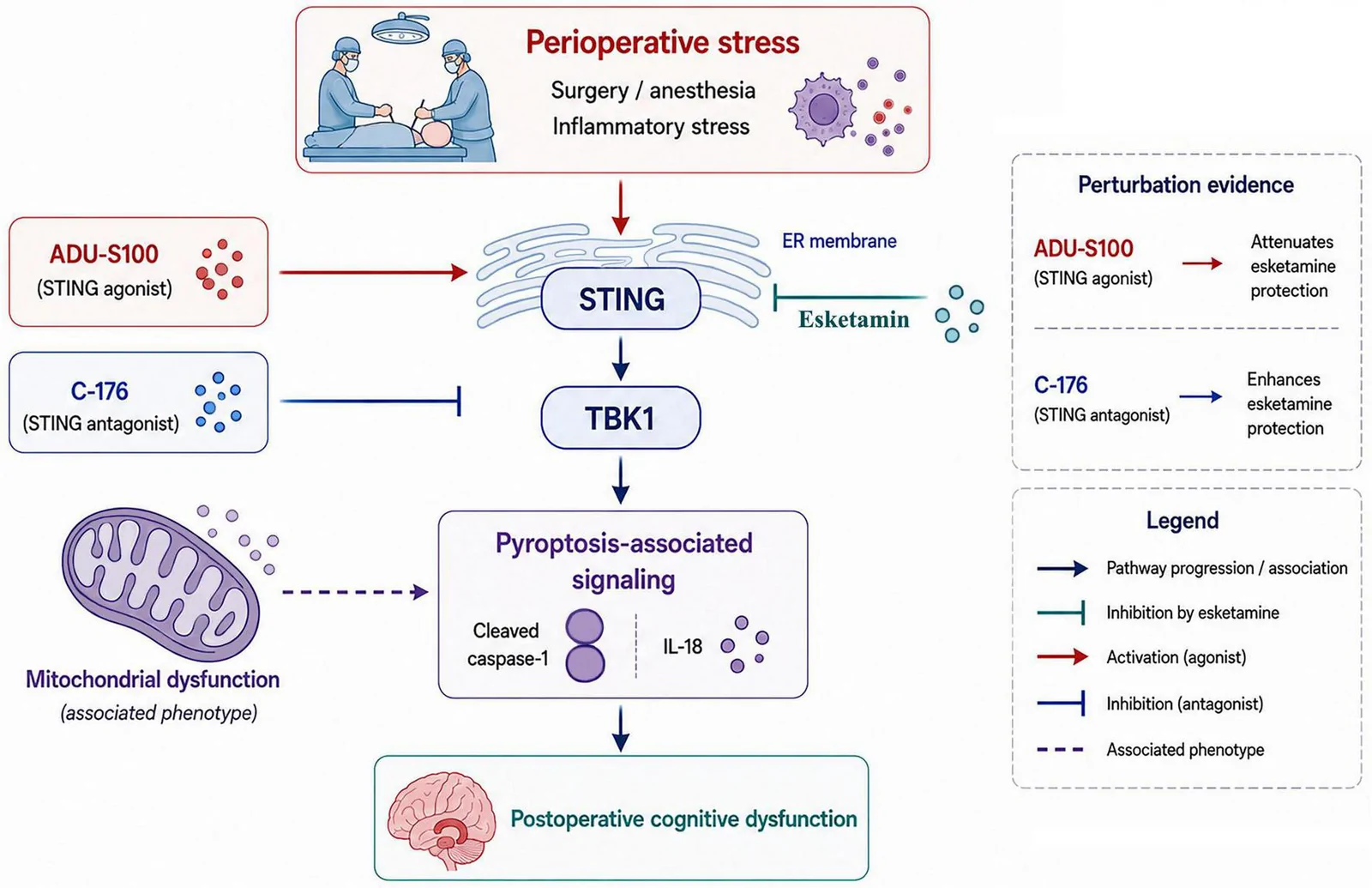
STING/TBK1-associated pyroptotic signaling and the modulatory role of esketamine in postoperative cognitive dysfunction. Perioperative stress activates STING/TBK1 signaling, which is associated with mitochondrial perturbation and pyroptosis-related responses, including caspase-1 activation, GSDMD cleavage, and IL-1β/IL-18 release. Esketamine attenuates excessive STING/TBK1 activation and reduces pyroptotic injury. Pharmacological perturbation supports pathway involvement, as the STING agonist ADU-S100 weakens, whereas the STING antagonist C-176 enhances, esketamine-mediated protection. Mitochondrial dysfunction represents an associated phenotype rather than an established direct target. Solid lines indicate direct perioperative evidence, whereas dashed lines indicate associative or contextual evidence.

The interaction of inflammasome signaling with other perioperative injury processes is also evident. In aged surgical mice, inhibition of NF-κB/NLRP3-associated inflammation occurred together with preservation of tight-junction proteins and reduced BBB permeability and cognitive impairment (Liu Q. et al., 2021). Such findings further support NLRP3 as part of a broader postoperative inflammatory network rather than an isolated upstream switch. These observations establish NLRP3 as an important component of the postoperative inflammatory landscape. They do not, however, demonstrate that NLRP3 is the principal molecular target through which esketamine exerts cognitive protection. Within the direct perioperative esketamine literature identified here, pathway-level evidence is currently stronger for TLR4/MyD88–p38, PARP1-related autophagic regulation, and STING/TBK1 signaling. NLRP3 is therefore more appropriately positioned as an interacting inflammasome node within the wider network rather than as the defining upstream mediator of esketamine action (see in Figure 2).

### 4.4 Microglial responses and BDNF–TrkB signaling

Microglia sit at an important interface between postoperative inflammation and neuronal plasticity. Although many experimental studies continue to describe microglial responses in terms of M1 and M2 polarization, these categories are better regarded as simplified descriptions of marker-defined inflammatory states rather than fixed cellular phenotypes.

Wen et al. investigated this relationship in aged rats with preoperative sleep disturbance, a vulnerability state that aggravated surgery-induced emotional and cognitive abnormalities. Sleep disturbance and surgery increased markers associated with a pro-inflammatory microglial response and disrupted BDNF–TrkB-related signaling. Esketamine attenuated these inflammatory changes, restored BDNF and TrkB-related signaling, and was accompanied by improved hippocampal synaptic plasticity and behavioral performance (Wen et al., 2024). The study therefore provides direct perioperative evidence linking esketamine exposure to coordinated changes in microglial inflammatory state, neurotrophic signaling, and synaptic function. However, no TrkB-specific antagonist or genetic manipulation was used to determine whether BDNF–TrkB signaling was required for the behavioral effect.

The biological connection between postoperative inflammation and neurotrophic dysfunction is supported by independent perioperative models. Microglial activity has been linked to complement-dependent loss of inhibitory synapses after surgery, with disruption of microglial or complement signaling improving postoperative cognitive performance (Tan et al., 2023). A different aged-mouse model showed that anesthesia and surgery were accompanied by NMDAR/Ca^2 +^/calpain overactivation, TrkB-FL truncation, BDNF–TrkB dysregulation, dendritic spine loss, and cognitive impairment; both memantine and the calpain inhibitor MDL-28170 attenuated these abnormalities (Qiu et al., 2020).

A complementary study focused on the balance between mature BDNF and proBDNF. Surgery and anesthesia reduced the hippocampal BDNF/proBDNF ratio and impaired dendritic structure and long-term potentiation in aged mice. Exogenous BDNF or inhibition of p75NTR improved synaptic plasticity and cognitive performance (Xue et al., 2022). These experiments establish neurotrophin signaling as a functionally relevant component of postoperative synaptic dysfunction, but they do not establish that the effect observed with esketamine in the Wen model is specifically TrkB-dependent.

BDNF–TrkB should therefore be positioned differently from the perturbation-supported TLR4/MyD88–p38 or STING/TBK1 branches. In the direct esketamine study, the pathway shows strong biological concordance with microglial and synaptic recovery, but pathway necessity remains untested.

Clinical findings further illustrate this distinction. In a randomized trial of 129 patients undergoing non-cardiac thoracic surgery, a single intraoperative dose of 0.5 mg/kg esketamine was associated with lower postoperative IL-6 and S100β and higher circulating BDNF (Luo et al., 2024). Nevertheless, CAM and MMSE outcomes did not differ significantly between groups. Peripheral BDNF therefore provides evidence of a treatment-associated biological response in this trial, not evidence that central BDNF–TrkB engagement mediated cognitive protection.

Within the present evidence framework, BDNF–TrkB is best regarded as a plausible link between regulation of the postoperative inflammatory environment and restoration of neuronal plasticity. Establishing it as a specific mediator of esketamine will require direct interruption of TrkB signaling within a perioperative cognitive model.

### 4.5 ROCK2/ADD1-associated remodeling and synaptic convergence

A major downstream point of convergence appears to be the preservation of synaptic structure and plasticity. Recent work has linked this functional endpoint to ROCK2/ADD1-associated remodeling in aged mice undergoing exploratory laparotomy under isoflurane anesthesia.

Proteomic screening identified Rho-associated coiled-coil kinase 2 (ROCK2) as a candidate molecule associated with the postoperative phenotype. Subsequent analyses showed increased ROCK2 expression and phosphorylation of α-adducin at Ser726 after surgery, both of which were attenuated by esketamine (Xu et al., 2026). These molecular changes occurred alongside recovery of dendritic spine density and complexity, synaptic ultrastructure, and electrophysiological measures of hippocampal plasticity. Springer records the study as Neurochemical Research volume 51, article 95, published in March 2026.

The strength of this study lies in the convergence of proteomic, molecular, structural, ultrastructural, electrophysiological, and behavioral observations. Its causal reach is more limited. Neither ROCK2 nor ADD1 was selectively inhibited, silenced, or genetically manipulated, so the study does not establish that disruption of the ROCK2/ADD1 branch determines the response to esketamine. The pathway should therefore be described as ROCK2/ADD1-associated remodeling, rather than as a proven mediator of esketamine action.

The importance of the synaptic phenotype itself is supported by other perioperative models. Hippocampal SIRT3 overexpression has been shown to improve dendritic organization and long-term potentiation together with postoperative learning and memory (Liu Q. et al., 2021). Separately, manipulation of mTOR-regulated autophagy restored synaptophysin and PSD-95 while improving memory after anesthesia and surgery (Gao et al., 2021). These studies arise from different upstream mechanisms, yet converge on structural or functional recovery at the synapse.

Evidence outside the perioperative setting also suggests that synaptic plasticity is a reproducible target of S-ketamine biology. In a postpartum-depression model, low-dose S-ketamine was associated with recovery of hippocampal synaptic features assessed using molecular markers, Golgi staining, transmission electron microscopy, and electrophysiological recording (Ren et al., 2022). Because this model does not reproduce perioperative cognitive injury, it provides cross-model plausibility rather than evidence for the ROCK2/ADD1 mechanism in POCD.

These findings make synaptic preservation a more defensible convergence point than any single upstream inflammatory pathway. Changes in PSD-associated proteins, dendritic morphology, ultrastructure, or long-term potentiation may reflect the combined consequences of inflammatory control, mitochondrial and autophagic homeostasis, neurotrophic support, and cytoskeletal remodeling. They should not be attributed automatically to ROCK2/ADD1, BDNF–TrkB, or another individual pathway without pathway-specific perturbation.

### 4.6 Indirect and supportive mechanisms

Several mechanisms emphasized in the broader esketamine literature remain biologically relevant but currently lack comparable direct validation in perioperative cognitive models. Their inclusion is useful for explaining biological plausibility, provided that they remain clearly separated from the direct perioperative evidence.

BBB disruption is one such mechanism. Experimental perioperative studies indicate that matrix metalloproteinases can contribute functionally to postoperative neurovascular injury. Genetic deficiency of MMP-9 reduced surgery-associated BBB disruption, neuroinflammation, and cognitive impairment in mice (Bi et al., 2017). In a later aged-mouse PND model, pharmacological inhibition of MMP-2 and MMP-9 restored claudin-5 and occludin expression, reduced glial activation, and attenuated postoperative cognitive decline (Ji et al., 2023). These findings support an MMP-dependent neurovascular contribution to perioperative cognitive injury. They do not, however, demonstrate that esketamine prevents POCD by inhibiting MMP-9 or directly restoring tight-junction proteins. BBB protection should therefore remain an upstream supportive mechanism rather than a core esketamine-specific branch.

A similar distinction applies to antioxidant signaling. In a rat POCD model, Nrf2/ARE pathway manipulation was examined together with hippocampal Nrf2/HO-1 expression, mitochondrial morphology, oxidative injury, and postoperative cognitive performance (He et al., 2023), supporting the relevance of this antioxidant system to perioperative brain stress. S-ketamine has also been linked directly to Nrf2/HO-1 activation and reduced oxidative injury in an experimental carbon tetrachloride-induced hepatic injury model (Xu et al., 2021). That study demonstrates an S-ketamine–Nrf2/HO-1 relationship, but in a non-neural and non-perioperative cognitive setting; it therefore cannot establish Nrf2/HO-1 as the mechanism of esketamine-associated cognitive recovery after surgery.

The α7-nicotinic acetylcholine receptor provides another example in which the perioperative pathway is biologically credible but the esketamine-specific link remains unproven. In aged rats subjected to anesthesia and surgery, α7-nAChR blockade with α-bungarotoxin partially reversed the cognitive, neuronal, microglial, and HMGB1/NF-κB-related improvements associated with electroacupuncture (Wang et al., 2021). This pharmacological reversal supports a functional role for α7-nAChR signaling in perioperative neuroinflammatory regulation. It does not demonstrate direct α7-nAChR engagement by esketamine.

Non-perioperative models provide additional mechanistic clues. In experimental traumatic brain injury, esketamine promoted AMPK/mTOR-dependent TFEB nuclear translocation, enhanced autophagic activity, and reduced oxidative neuronal injury; inhibition of AMPK attenuated several of these cellular effects (Tang et al., 2023). In cerebral ischemia/reperfusion, esketamine altered AKT signaling, autophagy, and marker-defined microglial response states, while the addition of rapamycin enhanced several protective effects (Gao et al., 2025). These studies strengthen the broader biological plausibility of autophagic, metabolic, and microglial regulation by esketamine, but neither disease context is interchangeable with postoperative cognitive dysfunction.

The distinction between supportive and direct evidence is therefore substantive rather than semantic. MMP-dependent BBB dysfunction, Nrf2/HO-1 signaling, α7-nAChR activity, AKT regulation, and related non-perioperative findings help define mechanisms that esketamine could plausibly engage. At present, they should not be used to infer that the same pathways mediate cognitive benefit in a surgery-related model unless direct perioperative perturbation evidence becomes available.

### 4.7 Integrated mechanistic framework

The direct experimental evidence favors a distributed model of esketamine action. Surgery and anesthesia generate overlapping inflammatory, metabolic, and cellular stress signals rather than engaging a single pathogenic cascade. TLR4/MyD88 signaling can recruit NF-κB- and p38-dependent inflammatory programs; PARP1-related autophagic regulation and SIRT3-associated mitochondrial pathways influence cellular stress tolerance; STING/TBK1 connects innate immune sensing with pyroptosis-associated signaling; and changes in microglial state can alter both inflammatory output and neurotrophic support. At the neuronal level, these processes converge on pathways governing cytoskeletal organization, dendritic stability, and synaptic plasticity.

The evidence within this network is not uniform. Pharmacological reversal provides comparatively strong support for TLR4/MyD88–p38 and STING/TBK1 signaling, while PARP1 perturbation strengthens the evidence for autophagic regulation. SIRT3 silencing provides additional mechanistic support at the cellular level, but this perturbation was not performed in the surgical animals. NF-κB, BDNF–TrkB, and ROCK2/ADD1 are currently supported mainly by direct perioperative associations or multimodal concordance rather than definitive necessity experiments. NLRP3, BBB/MMP-related regulation, Nrf2/HO-1, and α7-nAChR remain at a more contextual or supportive level.

The relative contribution of individual nodes is also unlikely to be fixed. Age, preoperative sleep disturbance, type and magnitude of surgery, anesthetic exposure, dosing and timing of esketamine, and pre-existing inflammatory or metabolic vulnerability may determine which mechanisms dominate in a given setting. Such context dependence may explain why different perioperative models identify overlapping but non-identical signaling pathways.

The available evidence is therefore most consistent with a distributed model in which esketamine modifies several interacting responses to perioperative brain stress. Inflammatory regulation, cellular stress homeostasis, glial–neuronal communication, and synaptic preservation provide plausible points of convergence, but their relative contribution is likely to vary across experimental conditions. Whether these mechanisms account for clinically meaningful benefit remains unresolved. The principal perioperative mechanistic studies and their level of experimental support are summarized in Table 1. Additional study-level details, including molecular readouts, pathway perturbation, and major interpretive limitations, are provided in Supplementary Table 2.

**TABLE 1:** Direct perioperative preclinical evidence for the proposed neuroprotective mechanisms of esketamine.

Study	Model	Mechanistic branch	Perturbation / rescue	Cognitive / functional outcome	Evidence interpretation
Xu et al., 2024	Aged mice; surgery	TLR4/MyD88-p38 MAPK	p38 activation with anisomycin attenuated esketamine effects	Improved spatial learning; reduced apoptosis/inflammation	Perturbation-supported; relatively strong
Li et al., 2022	Aged rats; exploratory laparotomy + cellular experiments	STING/TBK1-associated pyroptotic signaling	ADU-S100 weakened and C-176 enhanced esketamine-associated protection	Improved postoperative cognition; reduced pyroptosis-associated/cellular-stress abnormalities	Perturbation-supported; relatively strong
Zhao et al., 2025	Laparotomy mice + LPS-stimulated BV2 cells	PARP1/SIRT1-associated autophagic regulation	PARP1 overexpression; rapamycin comparison	Improved spatial learning; reduced inflammatory stress; restored autophagy-associated indices	Perturbation-supported; SIRT1 necessity not directly tested
Wang et al., 2026	Aged surgical mice + BV2 cells	SIRT3/AMPK/mTOR; mitochondrial homeostasis	SIRT3 silencing in parallel BV2 experiments	Improved cognition and mitochondrial/inflammatory indices	Mechanistically strengthened, but perturbation not performed in surgical animals
Han et al., 2026	Aged mice with preoperative sleep deprivation + surgery	TLR4/MyD88-NF-kB	No pathway-specific perturbation	Improved learning/memory with reduced microglial NF-kB-associated signaling	Direct perioperative association
Wen et al., 2024	Aged rats with preoperative sleep disturbance + surgery	Microglial response / BDNF-TrkB	No TrkB-specific blockade or genetic manipulation	Improved behavior and hippocampal synaptic plasticity	Direct perioperative association; mediation unproven
Xu et al., 2026	Aged mice; laparotomy under isoflurane	ROCK2/ADD1-associated remodeling	No selective ROCK2/ADD1 manipulation	Improved dendritic, ultrastructural and electrophysiological synaptic indices	Strong multimodal concordance; pathway necessity unproven
Hu et al., 2025	Perioperative model; transcriptomic discovery	Candidate genes (Ank1, Cbln4, L1cam, Gap43, Shh)	No functional validation	Exploratory molecular candidates	Hypothesis-generating

Evidence is ordered by mechanistic directness and perturbation strength rather than pathway popularity. NLRP3 is retained as contextual POCD/PND biology rather than a directly established esketamine-specific upstream mediator. Detailed molecular readouts, limitations, and DOI information are provided in Supplementary Table 2.

## 5 Evidence strength and clinical translation

The mechanistic evidence summarized in section “4 Multi-target neuroprotective mechanisms of esketamine in postoperative cognitive dysfunction” does not carry uniform inferential weight. Some perioperative studies have challenged candidate pathways experimentally, whereas others demonstrate concordant molecular, cellular, and behavioral changes without establishing pathway necessity. Clinical studies introduce a different problem: they offer greater translational relevance but vary substantially in surgical population, esketamine exposure, comparator regimen, cognitive construct, and duration of follow-up. Accordingly, the evidence is considered here according to mechanistic directness and clinical outcome definition, rather than simply whether an individual study reported a favorable result.

POD is treated as an acute and clinically relevant perioperative neurocognitive outcome but not as a surrogate for dNCR or longer-term postoperative cognitive decline. Likewise, isolated changes in MMSE or MoCA scores are not considered equivalent to formally defined postoperative neurocognitive disorders (Evered et al., 2018). This distinction is central to interpretation of the current clinical literature.

### 5.1 Strength of direct perioperative mechanistic evidence

The clearest mechanistic support comes from studies in which the proposed pathway was experimentally perturbed. Pharmacological activation of p38 MAPK attenuated the behavioral, histological, and anti-inflammatory effects of esketamine in aged surgical mice, providing functional support for the TLR4/MyD88–p38 branch (Xu et al., 2024). Similarly, activation of STING with ADU-S100 weakened esketamine-associated protection, whereas STING inhibition with C-176 enhanced it (Li et al., 2022). These reversal experiments provide stronger evidence of pathway involvement than parallel changes in signaling proteins and behavioral outcomes alone.

PARP1-related regulation of autophagy is supported by a different form of perturbation evidence. PARP1 overexpression reproduced inflammatory and autophagy-related abnormalities in BV2 cells, and esketamine partially reversed this phenotype; comparison with rapamycin further linked restoration of autophagic regulation to reduced inflammatory signaling (Zhao et al., 2025). These findings strengthen the case for PARP1-related cellular stress regulation, although they do not establish SIRT1 itself as a necessary mediator.

A second group of studies provides direct perioperative but predominantly associative evidence. In aged mice with preoperative acute sleep deprivation, esketamine suppressed TLR4/MyD88/NF-κB signaling in parallel with reduced hippocampal inflammation and improved cognition, but neither TLR4 nor NF-κB was selectively manipulated (Lin et al., 2024). Restoration of microglial BDNF–TrkB-associated signaling was likewise accompanied by improved synaptic plasticity and behavior without TrkB-specific blockade (Wen et al., 2024). ROCK2/ADD1-associated remodeling was supported by proteomic, structural, ultrastructural, electrophysiological, and behavioral observations, but neither ROCK2 nor ADD1 was selectively manipulated (Xu et al., 2026). These studies provide coherent multimodal evidence, although their causal reach remains lower than that of pathway-perturbation experiments.

Exploratory transcriptomic studies occupy an earlier stage of validation. Hippocampal transcriptome sequencing and network analysis identified Ank1, Cbln4, L1cam, Gap43, and Shh as candidate genes associated with esketamine treatment in a mouse PND model (Hu et al., 2025). Because these candidates emerged primarily from differential-expression and network analyses rather than pathway-specific perturbation, they are more appropriately regarded as hypothesis-generating targets than established mediators.

The preclinical literature therefore supports a multi-branch mechanistic model with unequal levels of validation. Perturbation-supported pathways currently provide the strongest mechanistic evidence, multimodal associations identify plausible intermediate mechanisms, and omics-derived candidates remain at the discovery stage.

### 5.2 Acute postoperative delirium as a distinct adjacent outcome

POD represents the most frequently evaluated acute neurocognitive endpoint in the direct clinical literature identified in this review. It should nevertheless be interpreted as a distinct postoperative syndrome rather than as evidence of POCD prevention. Several randomized trials have reported favorable results. In 356 older patients undergoing hip-fracture surgery, subanesthetic S-ketamine reduced the incidence of POD within 7 days from 29.8% to 9.6%; postoperative IL-1β, IL-6, and TNF-α concentrations were also lower in the intervention group (Hou et al., 2026). The trial used a 0.3 mg/kg loading dose followed by a 0.2 mg/kg/h infusion. A larger randomized trial of 372 older patients undergoing total hip or knee arthroplasty under neuraxial anesthesia reported POD within 3 days in 8.06% of patients receiving S-ketamine and 20.43% receiving placebo, corresponding to an adjusted odds ratio of 0.29 (95% CI 0.14–0.63) (Zhu et al., 2026). The restriction to a neuraxial-anesthesia setting is relevant when considering whether the result can be generalized to surgery performed under general anesthesia.

Positive findings have also been reported in cardiac surgical populations. In a triple-blind randomized trial of 140 patients undergoing off-pump coronary artery bypass grafting, intraoperative esketamine was associated with a lower 7-day POD risk (RR 0.474, 95% CI 0.231–0.970), accompanied by lower postoperative IL-6 and C-reactive protein concentrations (Ju et al., 2025). In another randomized trial involving cardiac valve surgery with cardiopulmonary bypass, POD occurred in 23.2% of patients receiving a single pre-induction dose of esketamine compared with 44.6% receiving placebo (RR 0.52, 95% CI 0.28–0.91) (Xiong et al., 2024). The analyzed population had a mean age of approximately 52 years, limiting direct extrapolation to older surgical cohorts. These favorable findings are counterbalanced by well-designed neutral trials. In 260 older patients undergoing total hip or knee arthroplasty, repeated perioperative low-dose esketamine did not significantly reduce POD during the first three postoperative days (8.5% vs. 10.8%), despite improvements in some pain and hemodynamic measures (Ma et al., 2024). A larger factorial randomized trial involving 426 patients undergoing elective noncardiac surgery likewise found no significant effect of a single 0.2 mg/kg dose of esketamine on POD or MMSE scores (Zhang et al., 2024). In that study, surgical start time rather than esketamine exposure was associated with POD incidence.

The two arthroplasty trials are particularly informative because broadly similar surgical populations produced different results. The neuraxial-anesthesia trial reported a substantial reduction in POD (Zhu et al., 2026), whereas the earlier trial using a repeated perioperative regimen found no significant benefit (Ma et al., 2024). This contrast argues against interpreting surgical category alone as a sufficient predictor of response. Some favorable effects also appear confined to the earliest postoperative period. In 108 older patients with fragile brain function undergoing thoracoscopic lung-cancer surgery, esketamine reduced POD at 24 h (3.8% vs. 15.1%), whereas the difference was no longer statistically significant at 72 h (0% vs. 7.5%) (Fu et al., 2025). IL-6 and S100β were also lower on postoperative day 1, but these peripheral biomarker changes do not establish mediation of the delirium effect.

Across these trials, esketamine or S-ketamine may reduce acute POD in selected surgical populations and perioperative regimens, but the effect is not consistent across randomized studies. Differences in anesthetic technique, surgical context, baseline vulnerability, dose, timing, and co-interventions may contribute to the observed heterogeneity. More importantly, a reduction in POD should not be interpreted as evidence that esketamine prevents dNCR or longer-term postoperative cognitive decline.

### 5.3 dNCR and longer-term postoperative cognitive outcomes

The evidence becomes more limited—and methodologically more heterogeneous—when attention shifts from acute delirium to postoperative cognitive decline itself. A recurrent pattern is that favorable signals are more often observed early after surgery, whereas evidence for persistent cognitive protection is less consistent.

Several trials in older gastrointestinal surgical populations illustrate this pattern. In 100 patients with preoperative anxiety undergoing gastrointestinal tumor surgery, a single 0.25 mg/kg dose of esketamine was associated with a lower study-reported cumulative incidence of dNCR within 7 days (26% vs. 54%) and higher MMSE scores on postoperative day 1 (Zha et al., 2025). IL-6 and S100β concentrations were also lower on day 1. The selected anxious population, short assessment window, and less rigorous dNCR definition limit comparison with trials using multidomain neuropsychological criteria.

A larger randomized double-blind study evaluated 153 older patients undergoing gastrointestinal tumor surgery. Esketamine was administered during both induction and maintenance, and cognition was assessed with a multitest neuropsychological battery at 7 and 30 days. The incidence of PND was lower at 7 days, whereas between-group cognitive differences were substantially less apparent by 30 days (Ma et al., 2025). The same trial reported lower postoperative IL-6, TNF-α, NSE, and Aβ1-42 concentrations, but these measurements were obtained peripherally and were not tested as mediators of the cognitive effect.

The most methodologically informative evidence comes from studies that incorporated multidomain assessment and correction for practice effects. In the randomized trial by Han et al., cognition was evaluated using a nine-test neuropsychological battery together with healthy controls for practice-effect correction. Esketamine reduced dNCR at 7 days (18.15% vs. 38.24%), whereas the difference in POCD at 3 months was not statistically significant (6.06% vs. 14.71%) (Han et al., 2023). The divergence between the early and 3-month results makes this study particularly important when interpreting claims of sustained cognitive protection.

Longer-term randomized evidence provides additional reasons for restraint. In 209 older patients undergoing major noncardiac surgery for malignant tumors, perioperative esketamine was associated with a signal of improved executive performance, but did not reduce POD or cognitive impairment at 90 days (Huang et al., 2024). Because esketamine was administered both intraoperatively and through postoperative patient-controlled analgesia, the trial also does not isolate a single exposure window.

Historical evidence with S(+)-ketamine points in the same direction. In a randomized open-heart surgery trial involving 106 patients, a formal neurocognitive battery was administered preoperatively and at 1 and 10 weeks. At 10 weeks, cognitive deficits involving at least two tests were present in 20% of the S(+)-ketamine group and 25% of the comparator group, a difference that was not statistically significant (Nagels et al., 2004). Thus, even an intervention with a biologically plausible neuroprotective rationale did not translate into a clear overall long-term neurocognitive advantage.

Not all studies labelled as dNCR or POCD provide equivalent evidence. A smaller randomized gastrointestinal-surgery trial reported a lower incidence of study-defined “DNR” with esketamine (16.13% vs. 38.71%), but the cognitive endpoint was operationalized using short-term postoperative MMSE rather than a multidomain, practice-effect-adjusted neuropsychological battery (Ma et al., 2023). It is therefore more appropriately regarded as definition-limited supportive evidence rather than evidence equivalent to formally defined dNCR.

The same caution applies to trials reporting isolated changes in screening scores. In a randomized study of modified radical mastectomy, S-ketamine was associated with better MMSE performance on postoperative day 1, but the difference was no longer apparent on day 2 (Zhang J. et al., 2023). Such short-lived score differences may indicate faster early recovery, but should not be described as prevention of POCD or durable postoperative neurocognitive disorder.

There is some supportive longer-term observational evidence, but its evidentiary weight is lower. A retrospective cohort of 140 older adults undergoing thoracic surgery reported lower study-labelled POCD and higher MoCA scores after intraoperative S-ketamine at 1 and 6 months (Wang et al., 2024). Because treatment allocation was nonrandomized and the cognitive definition and delirium ascertainment have methodological limitations, these findings are useful for hypothesis generation rather than as a counterweight of equal strength to randomized long-term trials. The final clinical audit likewise classified this study as longitudinal observational support rather than a primary causal anchor.

Intervention attribution introduces another source of uncertainty. In elderly patients undergoing lumbar spinal surgery, an esketamine–dexmedetomidine combination produced more favorable early cognitive findings than esketamine alone, together with lower NSE and S100β, but the absence of a conventional placebo arm prevents attribution of the effect specifically to esketamine (Tao et al., 2024). An opioid-free regimen combining dexmedetomidine and esketamine in older patients undergoing hip surgery similarly altered several perioperative outcomes, while delirium itself did not differ between treatment groups (Ye et al., 2024).

Regional-anesthetic combinations create the same problem. In a thoracoscopic-surgery trial, S-ketamine was administered together with thoracic paravertebral block rather than as an isolated intervention (Chen et al., 2022). The study remains clinically relevant, but any postoperative cognitive difference reflects the combined anesthetic strategy and cannot establish an esketamine-specific effect. The frozen screening set appropriately retained this type of study as direct clinical evidence while requiring lower attributional weight for the drug itself.

Overall, the available literature suggests a possible early cognitive benefit in selected populations, but does not establish durable protection against postoperative cognitive decline. The apparent attenuation of benefit with longer follow-up is clinically important, although the current heterogeneity in study design and outcome definition is too great to infer a definitive time-dependent treatment effect. The principal clinical studies are summarized in Table 2 according to population, esketamine exposure, neurocognitive outcome definition, assessment strategy, and timing. More detailed study-level information, including biomarker findings and methodological limitations relevant to interpretation, is provided in Supplementary Table 3.

**TABLE 2:** Key direct clinical studies of perioperative esketamine and postoperative neurocognitive outcomes.

Study	Population / *N*	Esketamine regimen	Outcome construct	Assessment / timing	Main finding	Key interpretation
Hou et al., 2026	Older hip-fracture patients; *n* = 128	Subanesthetic S-ketamine vs saline	POD	CAM; 7 d	Lower 7-d POD	Positive acute POD signal in a selected population.
Zhu et al., 2026	Older arthroplasty patients; *n* = 186	0.2 mg/kg S-ketamine vs. placebo	POD	3D-CAM; 7 d	8.06% vs. 20.43%; adjusted OR 0.29	Strong positive POD signal under neuraxial anesthesia.
Ju et al., 2025	OPCAB patients; *n* = 120	0.3 mg/kg esketamine vs. saline	POD	CAM-ICU; 7 d	Lower 7-d POD	Positive cardiac-surgery POD evidence.
Xiong et al., 2024	Older valve-surgery patients; *n* = 84	0.25 mg/kg esketamine vs. saline	POD	CAM-ICU; 7 d	Lower 7-d POD	Positive acute POD signal; does not establish long-term cognitive protection.
Ma et al., 2024	Older hip/knee arthroplasty; *n* = 260	Repeated low-dose esketamine vs. saline	POD	CAM; 7 d	No significant POD reduction	Important neutral RCT limiting universal prophylaxis claims.
Zhang et al., 2024	Older elective-surgery patients; *n* = 426	0.2 mg/kg esketamine vs. placebo (factorial RCT)	POD + screening cognition	CAM + MMSE; 7 d	No significant POD or MMSE benefit	Large neutral randomized counterweight.
Fu et al., 2025	Older lung-cancer surgery; fragile brain function; *n* = 124	0.25 mg/kg esketamine vs. saline	POD	CAM; 24 and 72 h	Lower POD at 24 h, not 72 h	Time-limited acute signal.
Zha et al., 2025	Older GI-tumor surgery with preoperative anxiety; *n* = 136	0.25 mg/kg esketamine vs. saline	POD + study-defined dNCR	CAM + MMSE-based cognitive assessment; ≤ 7 d	Lower POD/reported dNCR; higher early MMSE	Supportive early benefit; dNCR definition limits equivalence to formal multidomain dNCR.
Ma et al., 2025	Older GI-tumor surgery; *n* = 153	Esketamine vs. saline	Early PND/dNCR	Multidomain neuropsychological assessment; 7 and 30 d	Lower 7-d PND; differences attenuated by 30 d	Supports possible early benefit, not durable protection.
Huang et al., 2024	Older major non-cardiac tumor surgery; *n* = 196	Intraoperative esketamine + 48-h PCIA regimen	POD + 90-d cognition	CAM + neuropsychological follow-up; 90 d	Executive-recovery signal; no POD or 90-d cognitive-impairment reduction	Long-term benefit unproven; attribution is regimen-level.
Han et al., 2023	Older GI-tumor surgery; *n* = 136	Esketamine vs. placebo	dNCR + 3-mo POCD	Nine-test battery with practice-effect correction; 7 d and 3 mo	dNCR 18.15% vs. 38.24% at 7 d; 3-mo POCD difference not significant	Most informative early-positive/long-term-neutral cognitive pattern.
Ma et al., 2023	Older GI surgery; *n* = 80	Esketamine vs. control	Study-defined DNR	MMSE-based; early postoperative period	Lower reported DNR	Definition-limited supportive evidence; not equivalent to formal multidomain dNCR.

POD, postoperative delirium; dNCR, delayed neurocognitive recovery; PND, perioperative neurocognitive disorder; POCD, postoperative cognitive dysfunction; CAM, Confusion Assessment Method; 3D-CAM, 3-Minute Diagnostic Interview for CAM; MMSE, Mini-Mental State Examination; PCIA, patient-controlled intravenous analgesia. Short-term screening-score changes are not treated as equivalent to formally defined neurocognitive disorders. Detailed regimens, biomarkers, limitations, and DOI information are provided in Supplementary Table 3.

### 5.4 Biomarkers, attribution, and the translational gap

Peripheral biomarkers provide an apparent bridge between the experimental and clinical literatures, but their interpretive value is narrower than that of direct pathway measurements. Earlier clinical evidence has associated postoperative elevations in circulating IL-6– and S100β with cognitive decline (Peng et al., 2013), supporting their use as indicators of perioperative inflammatory or neural-stress responses rather than as evidence of a specific central mechanism.

This distinction is particularly important in the esketamine literature because biomarker changes frequently accompany favorable clinical outcomes. In the hip-fracture trial, lower POD incidence occurred together with lower IL-1β, IL-6, and TNF-α (Hou et al., 2026). The OPCAB trial similarly reported lower IL-6 and C-reactive protein alongside a reduction in POD (Ju et al., 2025), while the lung-cancer trial found lower IL-6 and S100β during a period in which the delirium difference was evident at 24 h but not at 72 h (Fu et al., 2025). These temporal and clinical differences make it difficult to infer that suppression of a particular circulating marker mediated the neurocognitive response.

The dNCR literature presents the same issue. Zha et al. reported lower IL-6 and S100β together with favorable early cognitive findings (Zha et al., 2025), whereas Ma et al. observed changes in IL-6, TNF-α, NSE, and Aβ1-42 in conjunction with a 7-day PND signal (Ma et al., 2025). Neither trial directly established that these peripheral changes corresponded to modification of the central inflammatory pathways identified in experimental models.

The randomized thoracic-surgery study provides an especially useful counterexample. A 0.5 mg/kg dose of esketamine lowered IL-6 and S100β and increased circulating BDNF, yet neither CAM nor MMSE outcomes differed significantly from placebo (Luo et al., 2024). The separation between biomarker modulation and cognitive outcome demonstrates why evidence of biological activity cannot be equated with clinical neuroprotection. The frozen clinical audit specifically classified this study as a biomarker–behavior discordance example rather than an efficacy anchor.

The translational gap is even wider when mechanistic evidence originates outside the perioperative cognitive setting. In experimental traumatic brain injury, esketamine has been linked to AMPK/mTOR-dependent TFEB translocation, autophagic regulation, and oxidative-stress reduction (Tang et al., 2023). Experimental cerebral ischemia/reperfusion studies provide additional evidence involving AKT signaling, autophagy, and microglial responses (Gao et al., 2025), while non-perioperative S-ketamine models support effects on synaptic plasticity (Ren et al., 2022). These findings strengthen biological plausibility but do not demonstrate that the same pathways mediate cognitive outcomes after surgery.

This distinction also applies to evidence derived from racemic ketamine, other enantiomers, and general POCD pathophysiology. Such studies can identify biologically credible links among inflammation, mitochondrial stress, autophagy, glial signaling, and synaptic function, but they occupy a lower level of directness than perioperative esketamine experiments. They should therefore inform interpretation of the direct evidence rather than increase its causal weight.

Human target engagement remains the principal missing link. Current clinical trials predominantly connect treatment exposure to peripheral biomarkers and cognitive phenotype; few directly demonstrate whether TLR4/MyD88–p38, STING/TBK1, PARP1-related autophagy, BDNF–TrkB, or other candidate pathways are engaged within the human central nervous system. A clinically useful mechanistic study would need to relate esketamine exposure, an appropriately validated central or CNS-proximal mechanistic readout, and a rigorously defined neurocognitive outcome within the same study rather than infer mediation from parallel postoperative changes.

Experimental models therefore provide increasingly detailed evidence that esketamine can modulate inflammatory, metabolic, glial, and synaptic processes, but the corresponding human evidence remains considerably less mechanistically resolved. Clinical efficacy is heterogeneous, durable cognitive protection has not been established, and peripheral biomarker modulation alone cannot close this gap. Esketamine should therefore be regarded as a biologically plausible but not yet established preventive therapy for perioperative neurocognitive disorders. The key translational question is which experimentally supported mechanisms are actually engaged in patients and whether such engagement predicts sustained cognitive benefit.

## 6 Discussion: remaining uncertainties and translational priorities

The central uncertainty is no longer the absence of plausible mechanisms, but the mismatch between mechanistic depth in experimental models and the heterogeneity of clinical neurocognitive outcomes. As summarized in Tables 1, 2, the apparent clinical signal depends strongly on what is measured and when, whereas only a subset of proposed mechanisms has progressed from association to experimental perturbation. These gaps define a more focused agenda for subsequent work.

### 6.1 Defining the clinical endpoint and its durability

A major obstacle to interpreting the clinical literature is that studies nominally addressing postoperative cognitive protection have not always measured the same phenomenon. POD captures an acute disturbance of attention and awareness; dNCR describes postoperative cognitive decline within a defined early recovery period; longer-term postoperative neurocognitive decline addresses a different clinical question altogether (Evered et al., 2018).

This distinction becomes particularly important when follow-up is extended. Several studies reviewed here suggest benefit during the early postoperative period, yet evidence for sustained cognitive protection is considerably less consistent. In the randomized trial by Han et al., cognition was assessed using a nine-test neuropsychological battery with correction for practice effects and predefined Z-score criteria. Esketamine was associated with less dNCR at 7 days, whereas the difference in POCD at 3 months was not statistically significant (Han et al., 2023). This early-positive but longer-term-neutral pattern is especially informative because it was observed using one of the more rigorous cognitive assessment strategies in the available esketamine literature.

Subsequent trials should make the intended cognitive construct explicit before enrollment and align the assessment strategy with that construct. Multidomain testing, baseline adjustment, prespecified diagnostic thresholds, and correction for practice effects are preferable when dNCR or persistent postoperative cognitive decline is the target outcome. Methodological work in postoperative cognitive assessment has long emphasized the influence of test selection, repeated testing, practice effects, and the choice of normative controls on the classification of postoperative cognitive decline (Rasmussen et al., 2001). More recent reviews of neuropsychological testing similarly support the use of appropriately selected multidomain batteries rather than reliance on an isolated screening score (Liu J. et al., 2021). Follow-up beyond hospital discharge is equally important. A recent meta-analysis of perioperative esketamine trials included 24 randomized studies but noted that follow-up was generally short, with several cognitive outcomes supported by low or very low certainty of evidence (Hung et al., 2025). If a study is designed to demonstrate neuroprotection rather than transient improvement in early recovery, cognitive outcomes at several weeks or months—and, where possible, measures of functional independence or return to preoperative activities—are more informative than a single early screening score.

A common framework of this kind would help answer a question that remains unresolved in the current literature: whether esketamine primarily modifies an acute perioperative brain state or can alter the subsequent trajectory of cognitive recovery.

### 6.2 From mechanistic association to human target engagement

Mechanistic breadth is no longer the principal limitation of the preclinical literature. The more important problem is uneven causal depth. Table 1 shows that some direct perioperative studies incorporated pathway perturbation, whereas others relied on parallel changes in molecular markers, cellular states, synaptic structure, and behavior. Pharmacological reversal provides comparatively strong support for the TLR4/MyD88–p38 branch (Xu et al., 2024) and STING/TBK1 signaling (Li et al., 2022), while PARP1 overexpression and autophagy-related intervention strengthen the evidence for PARP1-associated cellular stress regulation (Zhao et al., 2025). By contrast, the BDNF–TrkB findings remain based primarily on concordant microglial, neurotrophic, synaptic, and behavioral changes (Wen et al., 2024), and the ROCK2/ADD1 study provides extensive multimodal association without pathway-specific perturbation (Xu et al., 2026). Biological coherence is important, but it does not establish pathway necessity.

Future experiments would be more informative if pathway manipulation were incorporated into the same perioperative model in which postoperative cognition is measured. Selective inhibition, genetic manipulation, and rescue designs could distinguish processes that are required for protection from those that simply accompany it. Timing also matters. Molecular events measured hours after surgery may participate in inflammatory initiation, whereas changes observed days later may reflect adaptation, repair, or secondary synaptic remodeling. Establishing temporal order would therefore strengthen causal interpretation without requiring the field to continually add new candidate pathways.

The same problem applies to translation. Human studies still rely largely on peripheral inflammatory or neuronal-injury markers. Although circulating inflammatory markers have been associated with postoperative cognitive outcomes (Peng et al., 2013), such associations do not demonstrate activation of the corresponding pathway within the central nervous system. Reviews of perioperative biomarkers likewise emphasize the limited specificity of individual peripheral markers and the difficulty of using them to infer central neurocognitive mechanisms (Schaefer et al., 2019). This limitation is illustrated by the randomized thoracic-surgery trial in which esketamine lowered circulating IL-6 and S100β and increased BDNF, yet neither CAM nor MMSE outcomes differed significantly between treatment groups (Luo et al., 2024). Biomarker modulation in this setting therefore demonstrated biological activity without corresponding evidence of cognitive benefit.

Rather than expanding biomarker panels indiscriminately, mechanistic clinical studies should concentrate on a limited number of experimentally well-supported targets and link treatment exposure, target engagement, and cognitive phenotype within the same design. Serial sampling may clarify temporal relationships; mediation analyses can test whether biomarker changes lie on the pathway between treatment and outcome. In selected studies, cerebrospinal fluid sampling, neuroimaging, electrophysiological measures, or other CNS-proximal approaches may further narrow the gap between experimental and clinical evidence. A recent systematic review and meta-analysis identified associations between several CSF biomarkers and specific perioperative neurocognitive phenotypes, while also emphasizing substantial variation across PND subtypes and the need for further dNCR and longer-term POCD studies (Feng et al., 2025). The objective is not to reproduce rodent molecular biology in patients, but to determine whether the mechanisms supported experimentally are engaged at all in the perioperative human brain.

### 6.3 Exposure, patient susceptibility, and trial design

The intervention itself is also insufficiently standardized to support a clear dose–response or timing model. Clinical studies have used single-bolus administration, as in the gastrointestinal-tumor trial involving patients with preoperative anxiety (Zha et al., 2025), as well as combination regimens in which esketamine was administered with other active perioperative interventions (Tao et al., 2024; Ye et al., 2024). These differences are not trivial: they alter total drug exposure, the perioperative phase during which the brain is exposed, and the extent to which any cognitive effect can be attributed specifically to esketamine.

This attribution problem is particularly relevant when esketamine is combined with dexmedetomidine, regional anesthesia, opioid-sparing strategies, or postoperative patient-controlled analgesia. In the lumbar-spine trial, for example, the combination of esketamine and dexmedetomidine produced more favorable early cognitive findings than esketamine alone (Tao et al., 2024). In another study, opioid-free anesthesia combined esketamine with dexmedetomidine and several other perioperative components; delirium itself did not differ significantly between groups (Ye et al., 2024). Such regimens may be clinically attractive, but an observed improvement could reflect esketamine itself, reduced opioid exposure, better analgesia, greater hemodynamic stability, or interactions among these factors. Trials designed to isolate these components—or factorial designs when feasible—would provide more interpretable evidence than further comparisons of heterogeneous multimodal protocols.

Patient characteristics may also matter, but current between-study differences should not be mistaken for established treatment-response phenotypes. Preoperative anxiety was used to define a selected clinical population in one positive gastrointestinal-surgery trial (Zha et al., 2025), while preoperative sleep disturbance increased vulnerability in the direct perioperative experimental study of microglial and BDNF–TrkB-associated responses (Wen et al., 2024). Age, frailty, baseline cognitive reserve, mood symptoms, inflammatory burden, anesthetic technique, and surgical magnitude are all plausible modifiers. Whether any of them actually predicts benefit should be tested prospectively rather than inferred from the observation that positive and neutral trials enrolled different populations.

Safety belongs within the same exposure question. Psychotomimetic symptoms, dizziness, sedation, emergence characteristics, and cardiovascular effects should be reported systematically alongside cognitive outcomes, particularly in older and frail patients. These adverse-effect domains are pharmacologically relevant to perioperative esketamine and may vary with dose and co-administered agents (Mion and Himmelseher, 2024). The clinically relevant endpoint is ultimately a reproducible benefit–risk profile for a defined regimen in a defined population—not biological activity in isolation.

### 6.4 Limitations of the present evidence synthesis

This review should be interpreted within several constraints. It was designed as a structured narrative review with evidence mapping rather than a quantitative meta-analysis, and the available studies differ substantially in surgical population, esketamine exposure, cognitive definition, assessment instrument, and follow-up. A recent meta-analysis of 24 randomized esketamine trials likewise identified relatively short follow-up and low or very low certainty for several cognitive and biomarker outcomes (Hung et al., 2025). An earlier meta-analysis of adult surgical patients also pooled studies with substantial variation in cognitive endpoints and postoperative assessment times (Lin et al., 2024). The mechanistic literature remains dominated by animal and cellular models, and not all proposed pathways have been tested by perturbation or rescue. Indirect esketamine studies, ketamine/enantiomer research, and contextual POCD biology were therefore used to establish plausibility rather than to strengthen direct perioperative claims. Clinical evidence is likewise limited by variable outcome definitions, relatively few studies of persistent cognitive decline, and sparse evidence of central target engagement. Publication and selective-reporting bias cannot be excluded.

Progress will depend on tighter linkage between exposure, mechanism, cognitive phenotype, and time. A common neurocognitive framework, causal validation of the strongest mechanistic candidates, clinically interpretable target-engagement studies, and better-controlled treatment regimens would provide a firmer basis for determining whether esketamine has a durable role in perioperative neuroprotection.

## 7 Conclusion

Postoperative cognitive impairment arises from interacting inflammatory, metabolic, glial, and synaptic disturbances rather than from a single linear molecular cascade. Within this framework, the direct perioperative preclinical literature supports esketamine as a multi-target modulator, with evidence extending from inflammatory signaling and cellular stress homeostasis to glial responses and synaptic preservation. The strength of support, however, differs substantially across mechanisms. Some pathways have been strengthened by pharmacological or cellular perturbation, whereas others remain based on convergent molecular and functional associations. Accordingly, NF-κB- and inflammasome-related signaling should be viewed as components of a broader network rather than as a proven NF-κB/NLRP3/Caspase-1 master axis, and proposed effects involving the blood–brain barrier, antioxidant signaling, cholinergic regulation, or neurogenesis remain less directly established in perioperative esketamine models.

Clinical translation is less consistent than the experimental literature might suggest. Randomized studies provide evidence that esketamine may reduce POD or early postoperative cognitive decline in selected populations and treatment regimens, but clinically important neutral trials coexist with these findings, and evidence for sustained protection against longer-term cognitive decline remains limited. Esketamine should therefore not yet be regarded as an established preventive therapy for perioperative neurocognitive disorders. Its current significance lies in a biologically plausible, increasingly testable multi-target framework whose clinical value will depend on demonstrating reproducible cognitive benefit with standardized outcomes, appropriate follow-up, and direct linkage between treatment exposure and mechanism.
