# Supplementary material for: Neuroinflammation in postoperative cognitive dysfunction: the multi-target potential of esketamine in modulating microglial responses and synaptic integrity
Source: Front Mol Neurosci. 2026 Sep 2;19:1868647. doi: 10.3389/fnmol.2026.1868647 (PMC13579261; doi:10.3389/fnmol.2026.1868647)
Supplement: Supplementary file 1 [file Table_1.docx]

**Supplementary Table S1. Complete database-specific search strategies**

Databases were searched from inception with a literature eligibility cutoff of 1 May 2026. Searches were executed on 7 August 2026. Searches A and B formed the main evidence-selection track; Search C was used separately for broader POCD/PND pathophysiological context.

| **Database** | **Search** | **Purpose** | **Exact search strategy** | **Date handling** |
| --- | --- | --- | --- | --- |
| PubMed | A | Direct clinical/perioperative cognitive evidence | ("Esketamine"[Supplementary Concept] OR esketamine[Title/Abstract] OR "S-ketamine"[Title/Abstract] OR "S ketamine"[Title/Abstract] OR "(S)-ketamine"[Title/Abstract] OR "S(+)-ketamine"[Title/Abstract] OR "L-ketamine"[Title/Abstract]) AND ("Postoperative Cognitive Complications"[Mesh] OR "Emergence Delirium"[Mesh] OR "postoperative cognitive dysfunction"[Title/Abstract] OR POCD[Title/Abstract] OR "perioperative neurocognitive disorder"[Title/Abstract] OR "perioperative neurocognitive disorders"[Title/Abstract] OR "postoperative neurocognitive disorder"[Title/Abstract] OR "postoperative neurocognitive disorders"[Title/Abstract] OR "delayed neurocognitive recovery"[Title/Abstract] OR "postoperative cognitive decline"[Title/Abstract] OR "postoperative cognitive impairment"[Title/Abstract] OR "postoperative delirium"[Title/Abstract] OR ((perioperativ*[Title/Abstract] OR postoperativ*[Title/Abstract] OR surg*[Title/Abstract] OR anesthe*[Title/Abstract]) AND (cognit*[Title/Abstract] OR neurocognit*[Title/Abstract] OR delirium[Title/Abstract]))) AND ("1900/01/01"[Date - Publication] : "2026/05/01"[Date - Publication]) | Publication date ≤ 1 May 2026 |
| PubMed | B | Esketamine mechanistic evidence | ("Esketamine"[Supplementary Concept] OR esketamine[Title/Abstract] OR "S-ketamine"[Title/Abstract] OR "S ketamine"[Title/Abstract] OR "(S)-ketamine"[Title/Abstract] OR "S(+)-ketamine"[Title/Abstract] OR "L-ketamine"[Title/Abstract]) AND ("Neuroinflammation"[Mesh] OR neuroinflamm*[Title/Abstract] OR "Microglia"[Mesh] OR microglia*[Title/Abstract] OR "Blood-Brain Barrier"[Mesh] OR "blood-brain barrier"[Title/Abstract] OR "blood brain barrier"[Title/Abstract] OR "Inflammasomes"[Mesh] OR inflammasom*[Title/Abstract] OR NLRP3[Title/Abstract] OR "NF-kappa B"[Title/Abstract] OR "NF-κB"[Title/Abstract] OR NFKB[Title/Abstract] OR caspase-1[Title/Abstract] OR pyroptos*[Title/Abstract] OR "oxidative stress"[Title/Abstract] OR ROS[Title/Abstract] OR Nrf2[Title/Abstract] OR "heme oxygenase-1"[Title/Abstract] OR "HO-1"[Title/Abstract] OR "alpha7 nicotinic acetylcholine receptor"[Title/Abstract] OR "alpha 7 nicotinic acetylcholine receptor"[Title/Abstract] OR "alpha7-nAChR"[Title/Abstract] OR "α7-nAChR"[Title/Abstract] OR BDNF[Title/Abstract] OR TrkB[Title/Abstract] OR synap*[Title/Abstract] OR neurogenesis[Title/Abstract] OR PARP1[Title/Abstract] OR SIRT1[Title/Abstract] OR autophag*[Title/Abstract] OR MMP-9[Title/Abstract] OR "tight junction"[Title/Abstract] OR "tight junctions"[Title/Abstract] OR STING[Title/Abstract] OR TBK1[Title/Abstract] OR "p38 MAPK"[Title/Abstract] OR PI3K[Title/Abstract] OR AKT[Title/Abstract] OR GSK3*[Title/Abstract] OR SIRT3[Title/Abstract] OR AMPK[Title/Abstract] OR mTOR[Title/Abstract] OR ROCK2[Title/Abstract] OR ADD1[Title/Abstract] OR mitochondri*[Title/Abstract] OR mitophag*[Title/Abstract] OR "dendritic spine"[Title/Abstract] OR "dendritic spines"[Title/Abstract]) AND ("1900/01/01"[Date - Publication] : "2026/05/01"[Date - Publication]) | Publication date ≤ 1 May 2026 |
| PubMed | C | POCD/PND pathophysiology and contextual evidence | ("Postoperative Cognitive Complications"[Mesh] OR "postoperative cognitive dysfunction"[Title/Abstract] OR POCD[Title/Abstract] OR "perioperative neurocognitive disorder"[Title/Abstract] OR "perioperative neurocognitive disorders"[Title/Abstract] OR "postoperative neurocognitive disorder"[Title/Abstract] OR "postoperative neurocognitive disorders"[Title/Abstract] OR "delayed neurocognitive recovery"[Title/Abstract] OR "postoperative cognitive decline"[Title/Abstract] OR "postoperative cognitive impairment"[Title/Abstract] OR "surgery-induced cognitive dysfunction"[Title/Abstract] OR "surgery induced cognitive dysfunction"[Title/Abstract]) AND ("Neuroinflammation"[Mesh] OR neuroinflamm*[Title/Abstract] OR "Microglia"[Mesh] OR microglia*[Title/Abstract] OR "Blood-Brain Barrier"[Mesh] OR "blood-brain barrier"[Title/Abstract] OR "blood brain barrier"[Title/Abstract] OR "Inflammasomes"[Mesh] OR inflammasom*[Title/Abstract] OR NLRP3[Title/Abstract] OR "NF-kappa B"[Title/Abstract] OR "NF-κB"[Title/Abstract] OR NFKB[Title/Abstract] OR caspase-1[Title/Abstract] OR pyroptos*[Title/Abstract] OR "oxidative stress"[Title/Abstract] OR ROS[Title/Abstract] OR Nrf2[Title/Abstract] OR "heme oxygenase-1"[Title/Abstract] OR "HO-1"[Title/Abstract] OR "alpha7 nicotinic acetylcholine receptor"[Title/Abstract] OR "alpha 7 nicotinic acetylcholine receptor"[Title/Abstract] OR "alpha7-nAChR"[Title/Abstract] OR "α7-nAChR"[Title/Abstract] OR BDNF[Title/Abstract] OR TrkB[Title/Abstract] OR synap*[Title/Abstract] OR neurogenesis[Title/Abstract] OR PARP1[Title/Abstract] OR SIRT1[Title/Abstract] OR autophag*[Title/Abstract] OR MMP-9[Title/Abstract] OR "tight junction"[Title/Abstract] OR "tight junctions"[Title/Abstract] OR STING[Title/Abstract] OR TBK1[Title/Abstract] OR "p38 MAPK"[Title/Abstract] OR PI3K[Title/Abstract] OR AKT[Title/Abstract] OR GSK3*[Title/Abstract] OR SIRT3[Title/Abstract] OR AMPK[Title/Abstract] OR mTOR[Title/Abstract] OR ROCK2[Title/Abstract] OR ADD1[Title/Abstract] OR mitochondri*[Title/Abstract] OR mitophag*[Title/Abstract] OR "dendritic spine"[Title/Abstract] OR "dendritic spines"[Title/Abstract]) AND ("1900/01/01"[Date - Publication] : "2026/05/01"[Date - Publication]) | Publication date ≤ 1 May 2026 |
| Web of Science Core Collection | A | Direct clinical/perioperative cognitive evidence | TS=((esketamine OR "S-ketamine" OR "S ketamine" OR "(S)-ketamine" OR "S(+)-ketamine" OR "L-ketamine") AND ("postoperative cognitive dysfunction" OR POCD OR "perioperative neurocognitive disorder" OR "perioperative neurocognitive disorders" OR "postoperative neurocognitive disorder" OR "postoperative neurocognitive disorders" OR "delayed neurocognitive recovery" OR "postoperative cognitive decline" OR "postoperative cognitive impairment" OR "postoperative delirium" OR ((perioperativ* OR postoperativ* OR surg* OR anesthe*) NEAR/5 (cognit* OR neurocognit* OR delirium)))) AND PY=(1900-2026) | PY=1900–2026; records published/first available after 1 May 2026 excluded during screening |
| Web of Science Core Collection | B | Esketamine mechanistic evidence | TS=((esketamine OR "S-ketamine" OR "S ketamine" OR "(S)-ketamine" OR "S(+)-ketamine" OR "L-ketamine") AND (neuroinflamm* OR microglia* OR "blood-brain barrier" OR "blood brain barrier" OR NLRP3 OR inflammasom* OR "NF-kappa B" OR "NF-κB" OR NFKB OR caspase-1 OR pyroptos* OR "oxidative stress" OR ROS OR Nrf2 OR "heme oxygenase-1" OR "HO-1" OR "alpha7 nicotinic acetylcholine receptor" OR "alpha 7 nicotinic acetylcholine receptor" OR "alpha7-nAChR" OR BDNF OR TrkB OR synap* OR neurogenesis OR PARP1 OR SIRT1 OR autophag* OR MMP-9 OR "tight junction" OR "tight junctions" OR STING OR TBK1 OR "p38 MAPK" OR PI3K OR AKT OR GSK3* OR SIRT3 OR AMPK OR mTOR OR ROCK2 OR ADD1 OR mitochondri* OR mitophag* OR "dendritic spine*")) AND PY=(1900-2026) | PY=1900–2026; records published/first available after 1 May 2026 excluded during screening |
| Web of Science Core Collection | C | POCD/PND pathophysiology and contextual evidence | TS=(("postoperative cognitive dysfunction" OR POCD OR "perioperative neurocognitive disorder" OR "perioperative neurocognitive disorders" OR "postoperative neurocognitive disorder" OR "postoperative neurocognitive disorders" OR "delayed neurocognitive recovery" OR "postoperative cognitive decline" OR "postoperative cognitive impairment" OR "surgery-induced cognitive dysfunction" OR "surgery induced cognitive dysfunction") AND (neuroinflamm* OR microglia* OR "blood-brain barrier" OR "blood brain barrier" OR NLRP3 OR inflammasom* OR "NF-kappa B" OR "NF-κB" OR NFKB OR caspase-1 OR pyroptos* OR "oxidative stress" OR ROS OR Nrf2 OR "heme oxygenase-1" OR "HO-1" OR "alpha7 nicotinic acetylcholine receptor" OR "alpha 7 nicotinic acetylcholine receptor" OR "alpha7-nAChR" OR BDNF OR TrkB OR synap* OR neurogenesis OR PARP1 OR SIRT1 OR autophag* OR MMP-9 OR "tight junction" OR "tight junctions" OR STING OR TBK1 OR "p38 MAPK" OR PI3K OR AKT OR GSK3* OR SIRT3 OR AMPK OR mTOR OR ROCK2 OR ADD1 OR mitochondri* OR mitophag* OR "dendritic spine*")) AND PY=(1900-2026) | PY=1900–2026; records published/first available after 1 May 2026 excluded during screening |

**Notes.** PubMed searches used MeSH/Supplementary Concept terms together with Title/Abstract terms. Web of Science searches used the Topic field (TS) and publication-year filter. Because Web of Science does not support an exact day-level publication cutoff through PY, 2026 records were exported and records published or first available after 1 May 2026 were excluded during screening. Search C was not included in the main PRISMA-style flow.
