# Supplementary material for: Neuroinflammation in postoperative cognitive dysfunction: the multi-target potential of esketamine in modulating microglial responses and synaptic integrity
Source: Front Mol Neurosci. 2026 Sep 2;19:1868647. doi: 10.3389/fnmol.2026.1868647 (PMC13579261; doi:10.3389/fnmol.2026.1868647)
Supplement: Supplementary file 2 [file Table_2.docx]

**Supplementary Table S2. Detailed perioperative preclinical mechanistic evidence audit for esketamine**

| **Study** | **Model** | **Mechanistic branch** | **Primary action / readouts** | **Perturbation / rescue** | **Cognitive / functional outcome** | **Evidence strength** | **Manuscript interpretation** | **Manuscript ref. / DOI** |
| --- | --- | --- | --- | --- | --- | --- | --- | --- |
| Xu et al., 2024 | Aged mouse surgery-related cognitive model | TLR4/MyD88/p38 MAPK | Reduced TLR4/MyD88/p38 activity, cytokines and hippocampal apoptosis | LPS/anisomycin pathway challenge; p38 activation attenuated esketamine benefit | Morris water maze; CA1 injury/apoptosis | Perturbation-supported / High | Use as a principal direct inflammatory branch; avoid calling the pathway the sole mechanism. | [8] DOI: 10.1002/kjm2.12778 |
| Li et al., 2022 | 24-month SD rats; exploratory laparotomy + primary astrocyte model | STING/TBK1-associated pyroptotic signaling | Reduced STING/TBK1 activity, mitochondrial dysfunction and caspase-1/IL-18-associated pyroptotic changes | STING agonist ADU-S100 weakened protection; antagonist C-176 enhanced it | Open field, novel object recognition, Morris water maze; day-30 cognition | Perturbation-supported / High | Strong direct branch; pyroptosis findings must not be relabeled as proven NLRP3 mediation. | [7] DOI: 10.1016/j.brainresbull.2022.07.004 |
| Zhao et al., 2025 | C57BL/6J mouse exploratory laparotomy + BV2 LPS model | PARP1/SIRT1-related autophagic regulation | Lower PARP1, higher SIRT1, LC3/p62 and NAD+-related changes consistent with restored autophagic activity | PARP1 overexpression + rapamycin comparison; SIRT1 itself not selectively manipulated | Morris water maze; hippocampal inflammatory readouts | Perturbation-supported / High | Core direct branch requested by Reviewer 2; state PARP1/autophagy more strongly than SIRT1 necessity. | [9] DOI: 10.33549/physiolres.935577 |
| Wang et al., 2026 | Aged mice; abdominal exploratory laparotomy + LPS-stimulated BV2 cells | SIRT3/AMPK/mTOR and mitochondrial homeostasis | Reduced inflammation/oxidative stress, improved mitochondrial indices and microglial inflammatory-state markers | SIRT3 silencing attenuated multiple esketamine effects in parallel BV2 experiments | Morris water maze; hippocampal histology; cellular mitochondrial readouts | Cellular perturbation support / High-moderate | Retain as a direct metabolic/mitochondrial branch; explicitly state that SIRT3 silencing was performed in vitro. | [32] DOI: 10.36721/PJPS.2026.39.5.REG.15336.1 |
| Han et al., 2026 | 18-month mice; acute sleep deprivation + partial hepatectomy | TLR4/MyD88/NF-κB | Lower hippocampal TLR4/MyD88 and p-p65; reduced inflammatory cytokines; reduced p-p65 in Iba1-positive microglia | No pathway-specific rescue/knockdown | Open field + Morris water maze; hippocampal injury | Direct multimodal association / Moderate | Keep as direct NF-κB branch; wording should be 'associated with attenuation' rather than 'mediated by'. | [24] DOI: 10.3389/fnagi.2026.1798915 |
| Wen et al., 2024 | 24-month rats; preoperative sleep disturbance + exploratory laparotomy | Microglial inflammatory state and BDNF-TrkB signaling | Reduced pro-inflammatory microglial markers; restored BDNF/TrkB and synaptic-plasticity markers | No within-study TrkB antagonist/rescue | Cognitive/emotional behavior; dendritic and synaptic outcomes | Direct multimodal association / Moderate | BDNF-TrkB is a plausible candidate link, not proven mediator; avoid rigid M1→M2 claims. | [10] DOI: 10.1007/s12035-023-03860-4 |
| Xu et al., 2026 | Aged mice; exploratory laparotomy under isoflurane; proteomic + structural + electrophysiologic validation | ROCK2/ADD1-associated synaptic remodeling | Reduced ROCK2 and Ser726-ADD1 phosphorylation with recovery of dendritic structure, ultrastructure and electrophysiology | No ROCK2/ADD1-specific pharmacologic or genetic perturbation identified | Behavioral testing; dendritic spine density/complexity; electrophysiology | Direct multimodal association / Moderate | Present as ROCK2/ADD1-associated remodeling and synaptic convergence, not definitive mediation. | [41] DOI: 10.1007/s11064-026-04710-0 |
| Hu et al., 2025 | Mouse PND surgical model; hippocampal transcriptomics/WGCNA/PPI | Transcriptomic candidate genes/pathways | Identified Ank1, Cbln4, L1cam, Gap43 and Shh as candidate genes | Discovery/bioinformatic nomination without pathway-specific functional rescue | Morris water maze + Y-maze | Exploratory / Low | Use only for hypothesis generation; do not elevate candidate genes to established mechanisms. | [48] DOI: 10.2147/DDDT.S510752 |

*Note: Evidence is ordered by mechanistic directness and perturbation strength, not pathway popularity. NLRP3 is retained as contextual POCD biology rather than a directly established esketamine-specific upstream mediator. Association-only and discovery studies should not be described using necessity/mediation language.*
