# Supplementary material for: Neuroinflammation in postoperative cognitive dysfunction: the multi-target potential of esketamine in modulating microglial responses and synaptic integrity
Source: Front Mol Neurosci. 2026 Sep 2;19:1868647. doi: 10.3389/fnmol.2026.1868647 (PMC13579261; doi:10.3389/fnmol.2026.1868647)
Supplement: Supplementary file 3 [file Table_3.docx]

**Supplementary Table S3. Detailed clinical evidence audit of perioperative esketamine and postoperative neurocognitive outcomes**

| **Study** | **Design / population** | **N** | **Esketamine regimen / comparator** | **Outcome construct** | **Instrument / definition** | **Assessment timing** | **Main finding** | **Mechanistic biomarkers** | **Key interpretive limitation** | **Manuscript ref. / DOI** |
| --- | --- | --- | --- | --- | --- | --- | --- | --- | --- | --- |
| Hou et al., 2026 | Randomized controlled; older hip-fracture surgery | 356 randomized (178/178) | S-ketamine 0.3 mg/kg loading + 0.2 mg/kg/h infusion / Saline | POD | POD assessed through postoperative day 7; exact delirium instrument not specified in the accessible primary abstract | ≤7 d | POD 9.6% vs 29.8%; P<0.001 | IL-1β, IL-6, TNF-α; sleep quality | Large randomized sample; instrument/blinding details should not be over-interpreted beyond the report. | [50] DOI: 10.1007/s00210-025-04945-8 |
| Zhu et al., 2026 | Prospective randomized double-blind placebo-controlled; older THA/TKA under neuraxial anesthesia | 372 randomized (186/186) | S-ketamine 0.2 mg/kg IV over 1 h; S-ketamine also included in postoperative PCIA / Saline/placebo regimen | POD | POD within 3 d; 3D-CAM specified in the trial protocol/assessment framework | Postoperative days 1–3 | 15/186 (8.06%) vs 38/186 (20.43%); adjusted OR 0.29 (95% CI 0.14–0.63) | Pain/sleep/safety outcomes | Single high-volume neuraxial center; hallucinations, dizziness and nightmares were more frequent with S-ketamine. | [51] DOI: 10.1097/ALN.0000000000005800 |
| Zha et al., 2025 | Randomized placebo-controlled; older GI-tumor patients with preoperative anxiety | 100 analyzed (118 screened) | Esketamine 0.25 mg/kg at induction / Saline | POD + early cognitive change / dNCR-labelled | POD: Chinese 3D-CAM/CAM-S; cognition: MMSE d1–d7; cumulative dNCR reported | Postoperative days 1–7 | POD 24% vs 48%; cumulative dNCR 26% vs 54%; day-1 MMSE higher | IL-6; S100β | Early outcome only; dNCR construct is less rigorous than a multidomain practice-effect-adjusted neuropsychological definition. | [57] DOI: 10.2147/DDDT.S528902 |
| Ma X et al., 2025 | Randomized double-blind; older GI-tumor surgery | 153 analyzed (75 control/78 esketamine) | Esketamine 0.3 mg/kg induction + 0.3 mg/kg/h maintenance / Saline | dNCR/PND | Multitest neuropsychological battery; healthy-control change used in Z-score method; PND when ≥2 cognitive subtests crossed the prespecified threshold | 7 and 30 d | PND 8.97% vs 25.33% at 7 d; no significant between-group PND difference at 30 d | IL-6, TNF-α, NSE, Aβ1-42; PSQI | Single center; peripheral biomarker changes are correlates, not proof of central mechanistic mediation. | [58] DOI: 10.3389/fmed.2025.1653132 |
| Fu et al., 2025 | Randomized controlled; older lung-cancer patients with fragile brain function | 108 randomized; 106 analyzed (53/53) | Esketamine 0.25 mg/kg at induction + 0.1 mg/kg/h for 30 min / Saline | POD | Confusion Assessment Method (CAM), blinded assessment | 24 and 72 h | POD 3.8% vs 15.1% at 24 h (P=0.046); no significant difference at 72 h | IL-6; S100β | Selected vulnerable population; effect was confined to the earliest assessment. | [56] DOI: 10.1186/s12877-025-06268-y |
| Ju et al., 2025 | Prospective triple-blind RCT; off-pump CABG | 140 randomized | Esketamine 0.25 mg/kg/h intraoperative infusion / Saline | POD | CAM-ICU or 3D-CAM, twice daily during the first 7 postoperative days | ≤7 d | POD reduced; RR 0.474 (95% CI 0.231–0.970) | IL-6; CRP | Single tertiary center; biomarker findings are associative secondary endpoints. | [52] DOI: 10.1053/j.jvca.2025.04.013 |
| Ma C-B et al., 2024 | Prospective double-blind placebo-controlled RCT; older THA/TKA | 260 randomized/analyzed (130/130) | 0.20 mg/kg loading + 0.125 mg/kg/h infusion + 0.5 mg/kg in postoperative analgesia / Saline | POD | 3D-CAM twice daily; RASS used for subtype classification | Postoperative days 1–3 | POD 8.5% vs 10.8%; no significant difference | Hemodynamics, pain, sleep | Short 3-day follow-up; repeated perioperative exposure; no inflammatory biomarkers. | [54] DOI: 10.2147/DDDT.S477342 |
| Huang et al., 2024 | Randomized clinical trial; older major noncardiac tumor surgery | 209 | Esketamine 0.5 mg/kg intraoperative + 2 mg/kg in 48-h PCIA / Saline substitution | PND / longer-term cognitive outcome | Formal neuropsychological tests and TICS-m; POD and 90-day cognitive impairment assessed | Early postoperative + 90 d | Executive-function recovery signal, but POD and 90-day cognitive impairment were not reduced | Depression outcomes | Combined intraoperative and postoperative exposure prevents attribution to a single dose/timing window. | [59] DOI: 10.1097/MD.0000000000040028 |
| Xiong et al., 2024 | Triple-blind RCT; cardiac valve surgery with CPB | 116 randomized; 112 analyzed (56/56) | Esketamine 0.25 mg/kg before induction / Saline | POD + longitudinal MMSE | 3D-CAM or CAM-ICU twice daily for 7 d; MMSE at baseline, discharge, 30 d and 3 mo | POD ≤7 d; MMSE to 3 mo | POD 23.2% vs 44.6%; RR 0.52 (95% CI 0.28–0.91) | Delirium duration/subtype | Cardiac CPB population; positive POD effect should not be extrapolated to long-term POCD. | [53] DOI: 10.1213/ANE.0000000000006925 |
| Zhang et al., 2024 | Factorial randomized double-blind placebo-controlled noncardiac surgery trial | 426 analyzed | Esketamine 0.2 mg/kg / No esketamine | POD + MMSE | 3D-CAM on postoperative days 1, 3 and 7; MMSE secondary | Postoperative days 1, 3 and 7 | Esketamine did not reduce POD and did not improve MMSE | Time-of-day interaction | Factorial time-of-day design; a high-value neutral study that must be weighted equally with positive trials. | [55] DOI: 10.1097/JS9.0000000000001642 |
| Ma J et al., 2023 | Prospective double-blind RCT; older GI-tumor surgery | 68 enrolled; 62 analyzed (31/31) | 0.25 mg/kg loading + 0.125 mg/kg/h infusion / Saline | DNR/dNCR-labelled (definition-limited) | DNR operationalized from MMSE on postoperative day 3; POD assessed with RASS/CAM-ICU | Postoperative days 1–3 | DNR 16.13% vs 38.71%; POD was not the principal positive endpoint | Hemodynamics; pain | Short-term MMSE is not equivalent to a multidomain practice-effect-adjusted dNCR definition; small sample and attrition. | [61] DOI: 10.2147/DDDT.S406568 |
| Han C et al., 2023 | Randomized controlled; older GI surgery | 84 randomized; 67 analyzed + 15 healthy controls | Esketamine 0.15 mg/kg 5 min before surgery / Saline | dNCR + 3-month POCD | Nine-test neuropsychological battery; healthy-control practice-effect correction; predefined Z-score criterion across ≥2 tests | 7 d and 3 mo | Reported dNCR lower at 7 d (P=0.033); no significant POCD difference at 3 mo (P=0.247) | IL-6; S100β | Modest analyzed sample and attrition; strongest example of early-positive/long-term-neutral pattern. | [11] DOI: 10.2147/DDDT.S401161 |

*Note: POD, postoperative delirium; dNCR, delayed neurocognitive recovery; PND, perioperative neurocognitive disorder; POCD, postoperative cognitive dysfunction. Short-term MMSE/MoCA score changes and definition-limited DNR/dNCR labels are not treated as equivalent to multidomain, practice-effect-adjusted neuropsychological definitions. Peripheral biomarkers are interpreted as supportive correlates rather than proof of central mediation.*
